# Characterization of Extracellular Vesicles from Bronchoalveolar Lavage Fluid and Plasma of Patients with Lung Lesions Using Fluorescence Nanoparticle Tracking Analysis

**DOI:** 10.3390/cells10123473

**Published:** 2021-12-09

**Authors:** Magdalena Dlugolecka, Jacek Szymanski, Lukasz Zareba, Zuzanna Homoncik, Joanna Domagala-Kulawik, Malgorzata Polubiec-Kownacka, Malgorzata Czystowska-Kuzmicz

**Affiliations:** 1 Chair and Department of Biochemistry, Doctoral School, Medical University of Warsaw, Banacha 1, 02-097 Warsaw, Poland; magdalena.dlugolecka@wum.edu.pl; 2 Faculty of Chemistry, Warsaw University of Technology, Noakowskiego 3, 00-664 Warsaw, Poland; j.szymanski1997@gmail.com; 3 Chair and Department of Biochemistry, Medical University of Warsaw, Banacha 1, 02-097 Warsaw, Poland; lukaszzareba01@gmail.com (L.Z.); homoncik.zuzanna@gmail.com (Z.H.); 4 Department of Internal Medicine, Pulmonary Diseases and Allergy, Medical University of Warsaw, Banacha 1a, 02-097 Warsaw, Poland; jdomagala@wum.edu.pl; 5 Department of Surgery, Institute of Tuberculosis and Lung Diseases, Plocka 26, 01-138 Warsaw, Poland; m.polubiec@igichp.edu.pl

**Keywords:** fluorescence nanoparticle tracking analysis, extracellular vesicles, bronchoalveolar lavage fluid, plasma, non-small-cell lung cancer

## Abstract

The current lack of reliable methods for quantifying extracellular vesicles (EVs) isolated from complex biofluids significantly hinders translational applications in EV research. The recently developed fluorescence nanoparticle tracking analysis (FL-NTA) allows for the detection of EV-associated proteins, enabling EV content determination. In this study, we present the first comprehensive phenotyping of bronchopulmonary lavage fluid (BALF)-derived EVs from non-small cell lung cancer (NSCLC) patients using classical EV-characterization methods as well as the FL-NTA method. We found that EV immunolabeling for the specific EV marker combined with the use of the fluorescent mode NTA analysis can provide the concentration, size, distribution, and surface phenotype of EVs in a heterogeneous solution. However, by performing FL-NTA analysis of BALF-derived EVs in comparison to plasma-derived EVs, we reveal the limitations of this method, which is suitable only for relatively pure EV isolates. For more complex fluids such as plasma, this method appears to not be sensitive enough and the measurements can be compromised. Our parallel presentation of NTA-based phenotyping of plasma and BALF EVs emphasizes the great impact of sample composition and purity on FL-NTA analysis that has to be taken into account in the further development of FL-NTA toward the detection of EV-associated cancer biomarkers.

## 1. Introduction

In recent years, a lot of attention has been given to studies of extracellular vesicles (EVs) as a potential diagnostic and prognostic biomarker for many diseases including cancer. Since tumor-derived EVs can be found in large numbers in the biological fluids of cancer patients and their molecular cargo represents the tumor genotype and phenotype, they have undergone extensive research as a new variant of a liquid biopsy in cancer treatment [1].

In the case of non-small cell lung cancer (NSCLC), the bronchopulmonary lavage fluid (BALF) seems to be a good source of EVs from the tumor microenvironment [2]. BALF is currently extensively studied as a source of lung cancer-specific genetic or protein biomarkers [3]. Some reports suggest that BALF-derived biomarkers might be superior to serum biomarkers because they appear earlier during the cancer progression and at the higher concentrations [4]. The same can be true for BALF-derived EVs. Because of tumor proximity, EVs released by tumor cells may appear in BALF in the earlier disease stage and the higher concentration than in peripheral blood and reflect the tumor microenvironment more accurately. Therefore, a thorough study on the composition and function of BALF-EVs, representing EVs from the tumor microenvironment in NSCLC patients, can contribute to the development of biomarkers for patient therapy.

To develop clinically-viable EV-based diagnostic or prognostic screening assays, accurate and reproducible methods to evaluate the total concentration, size distribution, and single-particle phenotyping of EVs are urgently needed. Precise analysis of particles as small as EVs presents many technical challenges. In 1903, Prof. Richard Zsigmondy introduced the “Ultramicroscope”, which uses scattering light to visualize nanosized particles [5]. Thanks to this invention, many years later, dynamic light scattering (DLS) was developed [6]. It allowed for the calculation of the average size of nanosized particles but was unable to track individual particles simultaneously [7]. Within the last decade, nanoparticle tracking analysis (NTA) has emerged as the state-of-the-art method for the size and concentration characterization of exosomes and extracellular vesicles, overcoming the downfalls of the DLS method [7,8]. Particles are visualized by laser light, and the scattered light is recorded by a sensitive camera (CMOS/CCD) placed under the 90° angle to the irradiated plane [9]. This angle allows for the detection and tracking of the Brownian motion of particles sized from 10 to 1000 nm. Particles are detected, and their path is recorded. Using the Einstein–Stokes equation, the hydrodynamic diameter (size) of each particle present in the device’s cell unit is calculated [8,10]. Single NTA-based measurements in scatter mode allow for the quantification and size determination of nanosized particles, usually in the range of 40–1000 nm. However, they are unable to distinguish between EVs and other particles within their size range including protein aggregates, cell debris components, and lipoproteins [10]. Unfortunately, none of the currently available EV separation methods from biological fluids such as BALF or plasma is able to fully purify EVs from these contaminations. Therefore, the NTA-based scatter signal can only provide an estimation of the total particle number of EV-enriched fractions obtained from biological fluids. Recently, more advanced NTA instruments allowing for fluorescence detection have been developed. The number of membranous particles that likely represent EVs after staining with fluorescent lipophilic dyes or the number of specific exosomes or EV populations after fluorescent antibody staining for specific markers can be estimated in fluorescence mode [11].

Here, we have undertaken one of the first attempts at a comprehensive phenotypical characterization of BALF-EVs in comparison to plasma EVs. First, we characterized both EV types using established analytical methods such as western blotting, cryo-TEM, and bead-assisted flow cytometry, following the MISEV 2018 recommendations and standards [12]. Next, we performed NTA measurements in both scatter mode and fluorescence mode using the ZetaView device (Particle Metrix, Inning am Ammersee, Germany). The EVs were stained with the lipophilic dye Cell Mask Deep Red (CMDR, Thermo Fisher Scientific, Waltham, MA, USA) and fluorescence-labeled antibodies against some tetraspanins as typical exosome markers. Such analysis allowed us to determine the actual number and size of true EVs and investigate their composition in more detail (e.g., by determining the percentage of classical exosomes). Hereby, we developed an experimental setup based on fluorescence nanoparticle tracking analysis (FL-NTA) that can reveal the amount of bona fide EVs in isolates from heterogeneous particle solutions such as biological fluids.

## 2. Materials and Methods

### 2.1. Patients

The study group consisted of 34 patients (16 men and 18 women) consecutively enrolled with indications for BAL. The inclusion criterion was suspicion of lung cancer. During the diagnostic procedure, all patients were before anti-cancer treatment. Exclusion criteria involved contraindication to bronchoscopy, lack of patient agreement, ongoing anti-cancer treatment, immunosuppressive therapy, and infection. Patients were 42–80 years old, and the mean age was 66. Twenty-five patients were later confirmed with NSCLC after the diagnostic procedure. Three patients needed further diagnostics (two with no continuity of observation and one had the suspicion of NSCLC). Six patients turned out to have a different diagnosis than NSCLC including sarcoidosis, SCLC, and Pecoma cancer. The material (BALF and whole blood) was collected at the Institute of Tuberculosis and Lung Diseases in Warsaw from fasted patients. Ten mL of each patient’s whole blood was collected in vacuum blood collection tubes with EDTA (Vacutest Kima, cat. 13060, Arzergrande, Italy) and mixed. Within 1 h, the blood was transported at room temperature to the Medical University of Warsaw for plasma separation.

### 2.2. BAL-Procedure

BAL was performed according to recommendations of the Polish Respiratory Society [13] at the Institute of Tuberculosis and Lung Diseases in Warsaw. A 100 mL sample of saline (at body temperature) was injected in 20 mL doses via a bronchofiberoscope to the small bronchus, leading to the lesion affected by cancer (cBALF) and symmetrically to the same segment of the opposite lung (oBALF). The mean volume of recovered fluid was 30.5 mL ± 9.2 mL. The exclusion criteria for further BAL fluid analysis were: recovery fluid less than 30%, presence of more than 10% of epithelial cells, blood contamination, macroscopically visible mucus. After the BAL-procedure, BALF was transported at 4 °C within 1 h to the Medical University of Warsaw for further processing.

#### 2.2.1. Choosing EV Separation Method

For plasma EVs, we used centrifugation and homemade size-exclusion chromatography (SEC) columns and for BALF EVs, we chose differential ultracentrifugation as a suitable isolation method. The selection of isolation methods is described in detail in Section 4.1.

#### 2.2.2. Separation of EVs from Plasma of BAL Patients Using Homemade Mini-SEC Columns

Plasma was obtained from the patient’s whole blood sample by density centrifugation with the Lymphoprep™ (Stemcell, Köln, Germany) gradient as described before [14]. Briefly, about 5 mL of Lymphoprep™ was pipetted into a 15 mL tube, and the 5 mL of undiluted blood was carefully layered over the Lymphoprep™. The tubes were centrifuged (Eppendorf 5804R centrifuge and swing out rotor A-4-44, Hamburg, Germany) at 750× *g* for 30 min at room temperature (RT) with a disabled brake. After the centrifugation step, the upper layer of plasma was carefully aspirated with a Pasteur pipette to a new tube. After another centrifugation step (Eppendorf 5804R centrifuge and fixed-angle rotor F-45-30-11) at 2000× *g* for 10 min at RT, the supernatant was centrifuged again (Eppendorf 5804R centrifuge and fixed-angle rotor F-45-30-11) at 10,000× *g* for 30 min at 4 °C. Finally, the plasma was filtered using a 0.22-μm filter (qpore, PES-membrane, Heidelberg, Germany), aliquoted, and either stored frozen at −80 °C until further processing or directly used for EV-isolation. The homemade mini-SEC columns were prepared as described by Ludwig et al. [15] using Sepharose CL-2B (GE Healthcare, cat.17-0140-01, Chicago, IL, USA). Columns were stored at 4 °C filled with PBS (Gibco, cat. 70011-036, Invitrogen, Waltham, MA, USA, diluted with MiliQ water to 1×) with 0.05% sodium azide (Acros Organics, cat. 190381000, Antwerp, Belgium) as a preservative. Columns were reused up to three times. A 1 mL aliquot of the precleared and filtered plasma was thawed and applied to the mini-SEC column. After the sample entered the column, 2 mL of PBS (Lonza, Basel, Switzerland) was added, and 3 mL of void volume was collected (fractions 1–3, 1 mL each). Then, 4 mL of PBS was added, and EV-enriched fractions (1 mL each) were collected in separate tubes. EV fractions 5 and 6 were pooled (see Figure S1). Plasma EVs were either immediately analyzed or concentrated by centrifugation (Merck, Amicon^®^ Ultra-2 mL Centrifugal Filters, Darmstadt, Germany; Eppendorf 5804R centrifuge and swing out rotor A-4-44) at 4000× *g* for about 30 min at RT, and stored in 10 μL aliquots at −80 °C until further processing. The mean volume of the concentrated EV fraction was 111.6 ± 40.4 μL.

#### 2.2.3. Separation of EVs from BALF Using Differential Ultracentrifugation

BALF from the lung affected with either cancer or another lesion (cBALF) and from the opposite lung (oBALF) was strained through gauze and precleared by centrifugation (Eppendorf 5804R centrifuge and swing out rotor S-4-72) at 1000× *g* for 10 min at RT and then at 2500× *g* for 20 min at RT. Then, to break down the mucus, 2.5 mg of DTT (Sigma-Aldrich, Saint Louis, MO, USA, in water solution) was added, and the samples were shaken at 600 RPM 37 °C for 30 min. Afterward, samples were centrifuged (Beckman Coulter Optima XPN-80 Ultracentrifuge and SW32 Ti Swinging-Bucket rotor, Brea, CA, USA; Beckman Coulter tubes 355631) at 25,000× *g* for 40 min at RT. After that, the supernatant was collected and filtered using a 0.22 μm filter (Sartorius or GF, cellulose acetate double-membrane, Göttingen, Germany). Then, EVs were pelleted by ultracentrifugation at 110,000× *g* for 2 h at 4 °C (k_adj_ = 511.3). The EV-pellet was washed by ice-cold PBS and further centrifuged (Type 70.1 Ti Fixed-Angle Titanium Rotor; Beckman Coulter tubes 355603) at 110,000× *g* for 1 h at 4 °C (k_adj_ = 522.6). The EV-pellet was dissolved in PBS according to the starting BALF volume (at least 20 μL of PBS for every 1 mL of BALF) and stored in 10 μL aliquots at −80 °C until further processing. The mean volume of concentrated EV fraction was 77.0 ± 25.3 μL.

### 2.3. Immunocapture and Fluorescence Labeling of EVs for Flow Cytometry

An aliquot of BALF-EVs corresponding to 2 mL of BALF or 50 µL of nonconcentrated plasma-EVs (pooled fraction 5 and 6, 20 µg of protein) were bound to CD63, CD9, and CD81 coated Dynabeads (Invitrogen, cat. 10606D, 10620D, and 10622D, respectively) following the manufacturer’s protocol. Briefly, the EV sample volume was adjusted to 100 µL using isolation buffer (PBS with 0.1% BSA, filtered through a 0.22 µm filter). Then, 20 µL of Dynabeads were washed with isolation buffer and added to each EV sample. Samples were incubated ON with shaking (600 RPM) at 4 °C. The following day, the Dynabead-bound EVs were stained with either the specific markers (see Supplementary Table S1) or the isotype controls for 1 h at RT with mild shaking (600 RPM), then washed with isolation buffer and analyzed by flow cytometry.

### 2.4. Flow-Cytometric Analysis of EVs

Flow cytometry was performed on a BD FACSVerse 8 Color Flow Cytometer (BD, Franklin Lakes, NJ, USA) with BD FACSuite Software v.1.0.6. FCS files were then analyzed with FlowJo Software (LLC, Ashland, OR, USA). The stained bead-coupled EVs were resuspended in 150 µL PBS. A single-bead gate was set based on the FCS and SSC scatter and a minimum of 1500 beads were acquired. Gating strategies are shown in Figure S2.

### 2.5. Western Blotting of EVs

The protein content of the EVs separated from BALF, and the EVs concentrated from pooled fractions 5–6 from plasma was measured with the BCA Protein Assay Kit (Pierce, cat.23227) according to the manufacturer’s instructions. The EV amount corresponding to 100 µL of plasma or 4 mL of BALF was taken for SDS-PAGE. Samples were denatured for 5 min at 95 °C in reducing sample buffer (homemade). Proteins were separated on a 12% acrylamide gel and transferred into a nitrocellulose 0.2 µm membrane (GE Healthcare, Chicago, IL, USA), then blocked with either 5% non-fat milk(Sigma-Aldrich) or 5% BSA (Sigma-Aldrich) at RT for 1 h. Incubation with primary antibodies (recognizing Calnexin, Tsg101, Syntenin, CD9, or CD81, see Supplementary Table S1) was performed ON at 4 °C, followed by incubation with appropriate secondary HRP-conjugated antibodies (see Supplementary Table S1) for 2 h at room temperature. The chemiluminescence signal was achieved using the SuperSignal West Femto Maximum Sensitivity Substrate (Thermo Fisher Scientific, cat. 34095). Image acquisition was performed using a ChemiDoc Imager (Biorad, Hercules, CA, USA) with Image Lab Software (Biorad). Experiments were repeated at least three times.

### 2.6. Transmission Electron Microscopy

Imaging of EVs was conducted with the use of cryogenic transmission electron microscopy. Two to three µL of each sample (concentrated EVs from plasma, cBALF-EVs, and oBALF-EVs of one patient) were vitrificated in liquid ethane using the Thermo Fisher Scientific Vitrobot (blot time = 2 s, blot force = 0, blot total = 1) on TEM grids (Lacey Carbon or Quantifoil R2/2 copper, 200 mesh), previously glow-discharged (30 s, 25 mA) in a PELCO EasiGlow system. The grid freezing was conducted immediately before placing them into the Thermo Fisher Scientific cryo-electron microscope Glacios 200 kV in cryogenic conditions. For data analysis, EPU 2.7 software for single particle analysis and ImageJ software were used. For imaging, a Falcon3EC camera in linear mode without single-frame fractionation was used with a total electron dose per sample of 50 e/Å2, defocus −3.0 µm, −2.5 µm, −2.0 µm. For the Lacey carbon grid, a magnification of 72,000× with a pixel size 0.19 nm (1.9 Å) and of 52,000× with a pixel size 0.24 nm (2.4 Å) was used, and for the Quantifoil R2/2 grid, a magnification of 92,000× with pixel size 0.15 nm (1.5 Å) was used.

### 2.7. NTA-Scatter Measurement

EV size distribution profiles and concentration measurements in EV samples separated from BALF and plasma (nonconcentrated pooled fraction 5 and 6) were obtained using the ZetaView PMX220 (Particle Metrix) instrument equipped with a 488 and 640 nm laser and ZetaView 8.05.11 SP4 software. In accordance with the ZetaView manual, polystyrene 100 nm beads (Particle Metrix) were used for the daily calibration and instrument performance check. EV samples were diluted in PBS (Lonza) to obtain approximately 350 particles per frame. This concentration was chosen as an optimal concentration of EVs for the labeling experiments. The measurements in scatter mode were performed at RT at 11 positions in two cycles with the following settings for plasma and BALF-EVs—Sensitivity: 80, Shutter: 100, Minimal Brightness: 30, Trace length: 15, Min Area: 10, Max Area: 1000 nm/Class: 5, Classes/Decade: 64, Resolution: medium. The camera sensitivity was adjusted to also detect dim particles at a minimal background noise (measured in PBS). All settings were kept the same for all analyzed samples of a given sort (BALF-EVs, plasma-EVs) to minimize variability. At least three measurements of each sample were performed. For the daily calibration and reproducibility measurements, the following commercially available beads were used: (Particle Metrix, cat no 110-0020), YG488 beads (FluoSpheres™ Carboxylate-Modified Microspheres, Invitrogen, cat. no. F8803, lot.1835064), and DR660 beads (FluoSpheres™ Carboxylate-Modified Microspheres, Invitrogen, cat. no. F8807, lot.1893532). For all beads, the manufacturer’s dedicated settings for scatter measurements were used:

PS100 beads—Sensitivity: 60, Shutter: 100, Minimal Brightness: 30, Trace length: 15, Min Area: 10, Max Area: 1000 nm/Class: 5, Classes/Decade: 64, Resolution: medium.

YG488 and DR660 beads—Sensitivity: 60, Shutter: 100, Minimal Brightness: 30, Trace length: 15, Min Area: 5, Max Area: 1000 nm/Class: 5, Classes/Decade: 64, Resolution: medium.

The day-to-day repeatability and precision of the size and concentration measurements in scatter mode were quantified by performing daily measurements of the 100 nm PS100 beads and calculating the coefficient of variation (Supplementary Figure S3c,f).

#### 2.7.1. Fluorescent Labeling of EVs

For membrane labeling of EVs, the lipophilic membrane dye CMDR (Invitrogen) was used. The CMDR concentration for labeling was optimized experimentally on plasma-EVs (non-concentrated and concentrated) and BALF-EVs (see Supplementary Figure S4). The optimal final concentration for NTA, which ensured maximal EV-staining with a particle size corresponding to the size measured in scatter and a minimal background (only CMDR in PBS), was established as 4 ng/mL (Supplementary Table S1). The antibody concentration for tetraspanin-labeling and FL-NTA was adjusted by performing serial dilutions of antibodies in PBS and measurement in FL-NTA. The highest antibody concentration, which did not give high background, was chosen for each antibody as the optimal final dilution for the NTA measurement (Supplementary Table S1).

#### 2.7.2. Fluorescence-NTA

Prior to immunolabeling, all EV samples were measured in scatter mode to establish particle concentration. Before staining, predilution of EV sample/antibody/dye was prepared if needed. The EV-sample’s predilution was adjusted to achieve the highest concentration in range (about 350 particles per frame) for measurement in the scatter mode after final dilution post-labeling and differed according to the original concentration of a given EV sample. Fluorescence labeling was performed using prediluted EVs and prediluted antibody/CMDR in an approximately 9:1 ratio in a total volume of 10–50 µL for 2 h at RT in the dark. Then, the EVs were further diluted in PBS (usually 1:1000) and measured on NTA at RT at 11 positions in one cycle with the following settings:

For staining with antibodies:

F488, Sensitivity: 95, Shutter: 100, Minimal Brightness: 25, Trace length: 7, Min Area: 10, Max Area: 1000 nm/Class: 5, Classes/Decade: 64, Resolution: medium.

For staining with CMDR:

F640, Sensitivity: 91, Shutter: 100, Minimal Brightness: 25, Trace length: 7, Min Area: 10, Max Area: 1000 nm/Class: 5, Classes/Decade: 64, Resolution: medium. All immunolabeled samples were first evaluated in fluorescence mode with the function “low bleach” on, immediately followed by evaluation in scatter mode to minimize photobleaching. At least three measurements of each sample were performed.

For YG488 beads:

F488, Sensitivity: 80, Shutter: 100, Minimal Brightness: 20, Trace length: 7, Min Area: 5, Max Area: 1000 nm/Class: 5, Classes/Decade: 64, Resolution: medium.

For DR660 beads:

F640, Sensitivity: 80, Shutter: 100, Minimal Brightness: 20, Trace length: 7, Min Area: 5, Max Area: 1000 nm/Class: 5, Classes/Decade: 64, Resolution: medium.

The day-to-day repeatability and precision of the size and concentration measurements in fluorescence mode were quantified by performing daily measurements of the YG488 and DR660 beads and calculating the coefficient of variation (Figure S3a,b,d–f).

**Figure 1:**
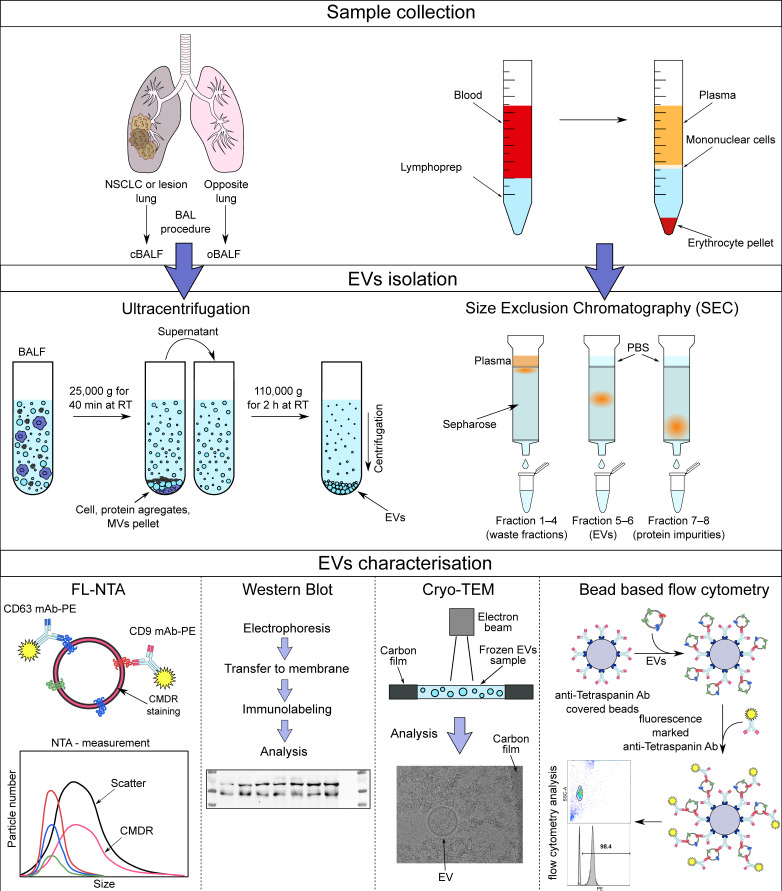
Summary of EV characterization methodology.

The isolation procedures and analysis methods of plasma and BALF-EVs used in this study are summarized in Figure 1.

### 2.8. Lysis of EVs

RIPA lysis buffer (Millipore, cat. no. 20-188, Merck) was used to lyse EVs obtained from the cell line NCI-H1975 (Hansa BioMed Life Sciences, cat. nr. HBM-NCl-H1975-100/5, Tallinn, Estonia). The 9 µL mixtures of EVs, after immunolabeling with CD9, CD63, CD81, and CMDR antibodies individually, were divided, and half of each was filled with PBS to the total volume of 1 mL to obtain optimal 1:1500 EV dilution. NTA-fluorescence was performed as described previously. The residues were incubated with 0.5 µL RIPA (10% solution) on ice for 30 min. After the lysis, PBS was added to the total 1 mL volume of each sample, giving the same optimal EV dilution, and FL-NTA measurements were repeated. The control sample was prepared in the same way, but RIPA was replaced with PBS for 30 min incubation on ice. Additionally, an appropriate dilution of RIPA in PBS was prepared to check whether RIPA solution alone interferes with NTA.

### 2.9. Subcellular Particles (Particularly Lipoproteins) Removal

Removal of subcellular particles was performed using the ExoQuick-LP for Lipoprotein Pre-Clear & Exosome Isolation Kit (System Biosciences, cat. no. EXOLP5A-1, Palo Alto, CA, USA) according to the manufacturer’s instructions with some modifications. Briefly, plasma was centrifuged at 2000× *g* for 10 min, RT, and then the supernatant was centrifuged at 10,000× *g* for 30 min at 4 °C. The supernatant was filtered through a 0.22 μm filter (qpore, PES-membrane). Subsequently, to remove any trace amounts of fibrinogen, thrombin from human plasma (Sigma-Aldrich, cat. no. 605190-100U-M) was added (final concentration 5 U/mL) and incubated for 5 min, RT, mixing gently. The supernatant was collected after centrifugation at 10,000 rpm for 5 min, RT, and 100 µL of the supernatant was added to beads prepared earlier according to the manufacturer’s instruction. Then, the sample was incubated for 3 h at 4 °C with rotation and then placed on a magnetic separator DynaMag-2 (Invitrogen, cat. no. 12321D) for 2 min, RT to remove bead-bound lipoproteins. The lipoprotein-cleared plasma sample was transferred into a new tube. Next, both the cleared plasma sample and control sample (plasma after only double centrifugation and filtration) were filled with PBS up to 1 mL and isolated using homemade mini-SEC columns, as described above. Next, the concentration of EVs from the cleared plasma sample (-LP) and control sample (CTRL) was measured by NTA. Finally, the diluted samples were labeled in the dark for 2 h at RT. Then, the FL-NTA measurements were performed.

### 2.10. Removal of Selected EV Populations by Immunomagnetic Isolation

We performed a magnetic separation of EV-subpopulations based on the expression of tetraspanins of a patient’s BALF-EVs sample with subsequent NTA measurements. BALF-EVs were isolated from the patient’s BALF using the method described above. CD63-specific (Invitrogen, Exosome-Human CD63 Isolation/Detection Reagent) and CD9-specific (Invitrogen, Exosome-Human CD9 Flow Detection Reagent) magnetic beads were washed with an assay buffer (PBS with 0.1% BSA), according to the manufacturer’s instructions using a magnetic rack (Invitrogen, DynaMag™-2 Magnet). In the next step, two separate samples for every bead-type consisting of 10 µL of the patient’s BALF-EVs sample, 90 µL of assay buffer, and 40 µL of the washed CD63 or CD9 specific beads, respectively, were prepared. The samples were mixed overnight (Topscien, TMM-5 Magic Mixer, Jiangshan, Ningbo, China) at 4 °C. The following day, the samples were spun for a few seconds and placed on the magnetic rack for 2 min to separate beads from the solution. Thereafter, the solutions were collected (unbound EV populations), and scatter and FL-NTA measurements were performed to detect CD63+, CD9+, and CMDR+ EVs. Furthermore, the residual magnetic beads were eluted from the adhered exosomes by incubation for 30 min at RT in 100 µL elution buffer (System Biosciences, Exo-Flow Elution Buffer). Subsequently, eluted magnetic beads were separated by placing the sample on a magnetic rack for 2 min. The residual fluid with exosomes was collected (eluted CD63+ and CD9+ populations) and underwent the same NTA measurements as samples from the unbound populations. A control sample represented the same unseparated patient-derived EVs. Additionally, we prepared a background control sample consisting of PBS instead of the patient’s EVs and 60 µL of both magnetic beads that underwent the same magnetic separation protocol.

### 2.11. Statistical Analysis

Statistical analysis was conducted using Excel (Microsoft, Redmond, WA, USA), GraphPad Prism (San Diego, CA, USA), and Statistica Software (StatSoft, Tibco, Palo Alto, CA, USA). The *p* value < 0.05 was considered significant. The quantitative analysis of data from NTA measurements was performed as follows:

Samples were measured in three repetitions. All samples were measured in scatter mode before and after labeling with dyes. Peak size (Mode–the value of size that appears most often in the collected statistical data analyzed by the Zeta View software) was chosen as the particle size in most samples. When the Mode could not be calculated by the instrument software, the Median (X50) size was taken for calculations. The mean concentration of EVs in the original sample was calculated and presented as the concentration of particles per 1 mL of plasma or 1 mL of BALF, respectively. The distribution of data was tested using the Shapiro–Wilk test. In the case of a normal distribution of the data, the paired two-tailed student’s *t*-test for dependent variables for comparisons of the concentrations and sizes of plasma EVs and BALF EVs was conducted. When there was no normal distribution of the data, the Wilcoxon matched pairs signed rank test for dependent variables was conducted. Nonparametric correlations of Spearman were calculated for associations between the concentration of particles and protein concentration.

### 2.12. EV-TRACK

Transparent Reporting and Centralizing Knowledge in Extracellular Vesicle Research (EV-TRACK) is an online crowdsourcing knowledgebase (http://evtrack.org, accessed on 2 February 2021) that centralizes EV biology and methodology intending to stimulate the authors, reviewers, editors, and funders to put experimental guidelines into practice. After uploading of the requested experimental parameters on the EV-TRACK platform, an EV-TRACK ID is assigned and an EV-metric is calculated. It is a feature designed to reflect the level of check-up in validation experiments and reporting of experimental parameters. It is presented as a percentage of fulfilled components from a list of nine, which were argued by the EV-TRACK consortium to be indispensable for unambiguous interpretation and independent replication of EV experiments [16,17].

We have submitted all relevant data of our experiments to the EV-TRACK knowledge base (EV-TRACK ID: EV200181). Our EV-metric is up to 63% for plasma and 67% for BALF-EVs of NSCLC patients.

## 3. Results

### 3.1. Characterization of Plasma/BALF EVs from NSCLC Patients

The process of selecting isolation methods and their detailed descriptions is described in the Materials and Methods section.

After the separation of EVs from plasma and BALF, we performed characterization experiments of the obtained EVs following MISEV guidelines [12].

**Figure 2:**
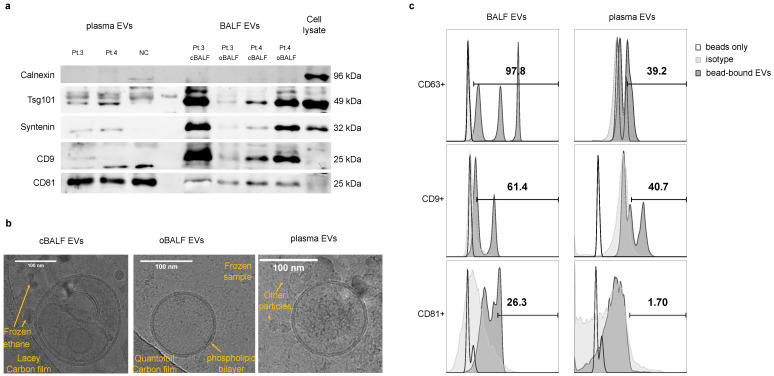
Characterization of plasma/BAL EVs from NSCLC patients. (**a**) Immunoblot analysis of EVs from plasma and BALF of two NSCLC patients (Pt.3, Pt.4), a normal donor (NC), and a cell lysate. The loaded EV amounts correspond to 100 μL of the patients’ plasma or 4 mL of the patients’ BALF, respectively. As a control, 10 μg of a cell lysate from the SEMK2 cell line was loaded. Full blots from (**a**,**b**) are provided in the Supplementary Materials. (**b**) Cryo-TEM imaging of EVs from cBALF, oBALF, and plasma from a NSCLC patient. (**c**) Flow cytometry of EVs from BALF and plasma attached to a mix of anti-CD63, anti-CD9, and anti-CD81 magnetic beads and then labeled with fluorescent anti-CD63, anti-CD9, and anti-C81 antibodies, respectively.

Figure 2 shows that the separation of EVs from both sources was effective. In Figure 2a, we can see that the exosomal markers Tsg101, CD9, CD81, and syntenin were detectable in both plasma-EVs and BALF-EVs. The non-EV marker calnexin is visible only in the cell lysate, which proves a good EV separation process and the lack of contaminating ER components. We checked the morphology of the separated EVs by cryo-TEM imaging (Figure 2b). In both BALF-EV samples, we found single round structures, comparing a clearly visible double-layer membrane with a thickness of 4 nm. The morphology and membrane thickness corresponded to the structures known as small EVs. Only a few such vesicles of 150 to 200 nm in size were visible per field, and only occasional clusters of EVs were seen. Most of the observed EVs were single EVs of a spherical shape. However, multi-vesicular particles with smaller double-membrane vesicles inside a bigger vesicle could also be observed such as in the presented cBALF-EV sample (Figure 2b). The much smaller, visible single-membrane dark irregular vesicles were not true vesicles, but frozen ethane as the solvent. In contrast, in the plasma-EV sample, there were many vesicles visible per field. However, only very few presented the typical size and double-membrane of true EVs. Most of the visible particles were single-layered, electron-dense, and had a smaller size than EVs (<50 nm). They mostly appeared in aggregates, and many of them displayed a typical striped inside structure. Based on previously reported cryo-TEM analyses of EV preparations, we concluded that these particles are lipoproteins and protein aggregates (especially the stripped structures, typical for lipoproteins; see also the cryo-TEM picture at smaller magnification in Figure S7b). Bead-assisted flow cytometry analysis of the presence of tetraspanin at the EV surface confirmed the results obtained by western blotting. BALF EVs captured by tetraspanin beads showed a higher percentage for all three exosomal markers (97.2% CD63, 61.4% CD9, and 26.3% CD81 positive particles) than plasma EVs (39.2% CD63, 40.7% CD9, and 1.7% CD81 positive particles). The supplementary data for gating strategy and flow cytometry analysis of the single bead type with BALF EVs are shown in Figure S2.

### 3.2. NTA of Plasma and BALF EVs in Scatter Mode

**Figure 3:**
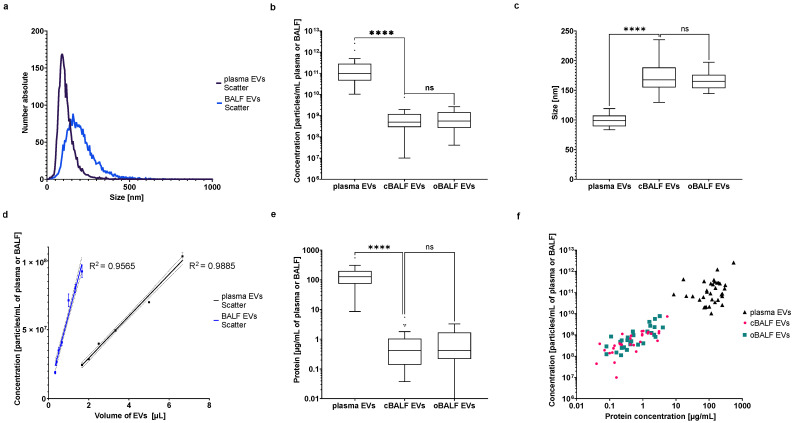
NTA of plasma and BALF EVs in scatter mode. (**a**) Distribution of number and size of detected particles in scatter mode of plasma and BALF-EVs. A representative NTA histogram of one patient is shown. (**b**) Mean concentration of EVs from plasma, cBALF, and oBALF for all patients. (**c**) Mode size of EVs from plasma, cBALF, and oBALF for all patients. (**d**) The concentration of particles measured in scatter mode depend on the volume of EV sample taken for the measurement. A line from simple linear regression with CI and R^2^ was plotted for both types of EVs. (**e**) Protein concentration calculated for one mL of plasma or BALF. (**f**) Correlation between the concentration of particles and protein concentration for plasma and BALF-EVs. Graphs (**b**,**c**,**e**) present a Tukey plot for all patients. **** refers to *p* value ≤ 0.0001, ns refers to *p* value > 0.05 from the Wilcoxon test-paired comparison (**b**,**e**) and *t*-test-paired comparison (**c**).

Our NTA-analysis in scatter mode of EVs separated from plasma and BALF showed a different particle distribution of both EV types. A representative particle size distribution of plasma and BALF EVs from one patient is shown in Figure 3a. The BALF-EVs present a broader size distribution and are less numerous than plasma EVs from the same patients. The pairwise comparison of EVs isolated from all analyzed patients revealed that the concentration of plasma-EVs (mean ± SD: 2.44 × 10^11^ ± 4.71 × 10^11^ particles/mL of plasma) was significantly higher than the concentration of BALF EVs (mean ± SD: cBALF: 8.85 × 10^8^ ± 1.30 × 10^9^; oBALF 1.22 × 10^9^ ± 1.68 × 10^9^ particles/mL of BALF) for all patients (Figure 3b). Our identified mean total particle numbers in plasma corresponded very well to the mean particle amounts detected by Mork et al. directly in platelet-free plasma (PFP) of fasting NC by NTA (Nanosight LM10 instrument), with a comparable broad intra-individual variation (observed range 8.9 × 10^10^–1.0 × 10^12^, 95% reference interval 1.4 × 10^11^–1.2 × 10^12^, Mork 2017). There was no significant difference between the concentration of EVs in cBALF and oBALF. The mode size of EVs from plasma (mean ± SD: 98.43 ± 10.13 nm) was bigger than the detected particle mean size in PFP (62 nm) by Mork et al., but comparable to the particle mean size of postprandial samples (93 nm) [18]. Our detected mode size of plasma particles was significantly smaller than the mode size of BALF-EVs (mean ± SD: cBALF: 171.95 ± 23.72 nm; oBALF 166.60 ± 13.82 nm) for all patients (Figure 3c). There was no significant difference between the mode size of EVs in cBALF and oBALF.

To check the NTA-measurement linearity in scatter mode, we performed measurements of different EV amounts. The results (Figure 3d) showed that there was a linear correlation (R^2^ for BALF EVs was 0.9565 and for plasma EVs 0.9885) between the EV amount and NTA-signal in scatter mode. Protein concentration of plasma EVs (mean ± SD: 147.45 ± 105.99 µg/mL of plasma) was significantly higher than the concentration of BALF-EVs (mean ± SD: cBALF: 0.91 ± 1.21; oBALF 0.99 ± 1.06 µg/mL of BALF) for all patients (Figure 3e) and was comparable to EV-protein concentrations obtained by other researchers using the same isolation methods (e.g., by Dong et al., who reported a concentration of 160.27 ± 14.81 µg/mL [19]). Our obtained BALF-EV protein concentrations were in a similar range as concentrations for EVs obtained by ultracentrifugation from cell culture or urine by Dong et al., but significantly lower than for BALF-EVs isolated from cystic fibrosis, asthmatic, and primary ciliary dyskinesia patients by Rollet-Cohen et al. [20]. In this case, the authors obtained EV protein concentrations from around 43 µg/mL for asthma to around 158 µg/mL. However, the difference may be due to differences in pre-analytical handling, isolation method (no wash-step after EV pelleting like in our case), and a different patient cohort. In our study, there was no significant difference between the protein concentration of EVs in cBALF and oBALF. For both BALF EV types, there was a meaningful correlation between the concentration of particles on NTA and protein concentration (Spearman correlation r_s_ = 0.82 for cBALF EVs and r_s_ = 0.77 for oBALF EVs, *p* < 0.0001) (Figure 3f). In plasma EVs, a similar correlation between those two factors was lacking (Spearman correlation r_s_ = 0.26 for plasma EVs, *p* > 0.05).

### 3.3. Membrane Labeling of Plasma and BALF EVs

**Figure 4:**
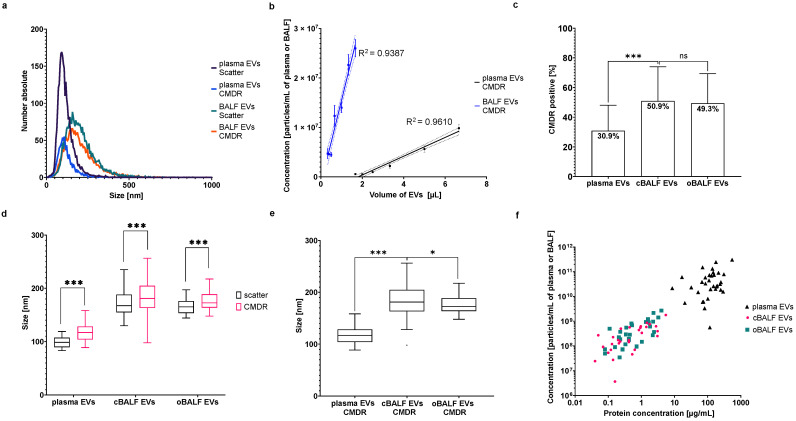
CMDR labeling of EVs. (**a**) Distribution of particles in scatter and fluorescent mode (640 nm) for EVs from plasma and cBALF. A representative NTA histogram of one patient is shown. (**b**) Concentration of particles measured in fluorescent mode (640 nm) depending on the volume of the EV sample taken to measure the BALF and plasma EVs of one patient. A line from simple linear regression with CI and R^2^ was plotted for both types of EVs. (**c**) The percent of CMDR-positive particles in comparison to all particles visible in scatter mode for all analyzed EV types. (**d**) Comparison of EV mode sizes measured in scatter and after CMDR labeling within the three analyzed EV types. (**e**) Comparison of the mode sizes measured after CMDR labeling of the three analyzed EV types. (**f**) Correlation between the concentration of particles and protein concentration for plasma and BALF-EVs after CMDR staining. Graph (**c**) presents mean and SD for all patients. Graphs (**d**,**e**) presents a Tukey plot for all patients. *** refers to *p* value ≤ 0.0002, * refers to *p* value ≤ 0.05, ns refers to *p* value > 0.05 from *t*-test-paired comparison.

The size distribution of particles after CMDR labeling (final concentration during NTA measurement 4 ng/mL CMDR, see Supplementary Figure S4) for both EV types (plasma and BALF) in FL-NTA was similar to scatter mode and is represented in Figure 4a. Concentration measurements of different EV volumes after CMDR labeling in fluorescent mode (640 nm) also showed linear dependency as it was in scatter (Figure 4b). R^2^ for BALF-EVs in CMDR was 0.9387 and for plasma EVs 0.9610. Labeling of EV samples with CMDR revealed that BALF-EVs had a significantly higher percent of particles positive for CMDR (cBALF 50.9% and oBALF 49.3%) than plasma EVs (30.9%) (Figure 4c). There was no meaningful difference between the percent of CMDR positive particles in cBALF-EVs and oBALF-EVs. The mode size of EVs positive for CMDR was significantly higher than in scatter mode for all EV types (Figure 4d). However, plasma-EVs were still significantly smaller (mean ± SD: 117.32 ± 17.93 nm) than both BALF-EV types (mean ± SD: cBALF: 183.23 ± 32.70 nm; oBALF 175.80 ± 17.01 nm), and interestingly, cBALF-EVs were meaningfully larger than oBALF-EVs in the CMDR staining (Figure 4e). Similar to the scatter mode measurement, for both BALF-EV types, there was a correlation between the concentration of particles and protein concentration in FL-NTA at 640 nm (Spearman correlation r_s_ = 0.74 for cBALF-EVs and r_s_ = 0.72 for oBALF-EVs, *p* < 0.0001) (Figure 4f). There was no significant correlation between these two factors (Spearman correlation r_s_ = 0.23, *p* > 0.05) in plasma-EVs.

### 3.4. Antibody Labeling of Plasma, BALF, and NSCLC Cell Line EVs

**Figure 5:**
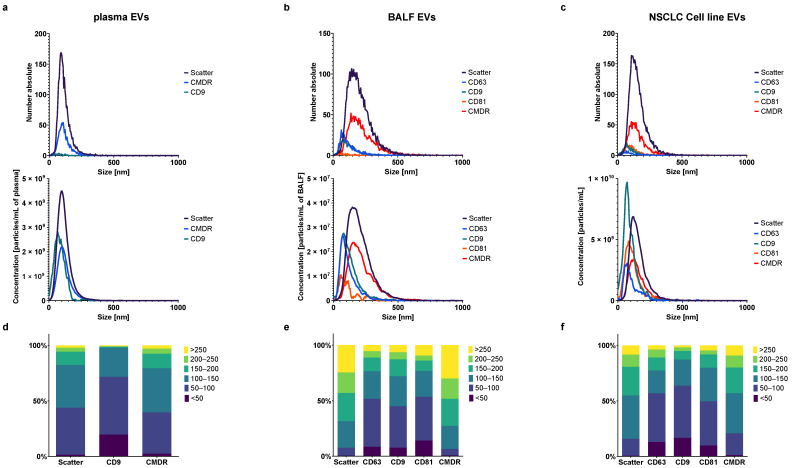
Size distributions from FL-NTA of particles in scatter and fluorescent mode (488, 640 nm) after immunolabeling of EVs against EV specific markers CD63, CD81, and CD9 and membrane marker CMDR. (**a**–**c**) Representative distributions measured in scatter and fluorescence mode of EVs from plasma (**a**), oBALF (**b**), and NSCLC cell line (**c**). The upper graphs present absolute numbers of particles measured by NTA, and the lower present the concentration of particles per one mL of plasma/BALF/NSCLC cell line EVs. The concentration of particles per one mL of each biological fluid is calculated by Particle Matrix software as Concentration = Number/(Area × depth). The component “Area x depth” differs between measures and is constant in the single measurement. Its value depends on outliers during each measurement. This “measured volume” effect leads to a different curve profile between the number absolute graphs and concentration graphs. (**d**–**f**) Concentration of particles—fraction of all particles [%] in six size fractions (<50 nm, 50–100 nm, 100–150 nm, 150–200 nm, 200–250 nm, >250 nm) for plasma (**d**), BALF (**e**) and cell line EVs (**f**). For all exact percent values, see Supplementary Table S4.

After fluorescence staining against typical exosomal tetraspanins (CD63, CD9, CD81), the plasma-derived EVs showed a very different profile in FL-NTA in comparison to BALF-EVs and cell line-derived EVs (cl-EVs), which were more similar to each other (Figure 5). The measured signal from the exosomal markers CD63, CD81, and CD9 was very weak and mostly below the detection limit for plasma-EVs. Only in a few plasma-EVs samples (three out of 34), the signal from CD9 was detectable, but much lower than in scatter or CMDR (Figure 5a). In BALF-EVs, in most cases, all tetraspanin-positive EVs were well detectable, though their distribution explicitly shifted toward smaller sizes (Figure 5b). Antibody labeling against tetraspanins of commercially available standard EVs derived from the NSCLC cell line (cl-EVs) showed similar results, albeit the particle size distribution was slightly narrower (Figure 5c). A closer analysis of the size distributions after dividing the particles into six size fractions provided more differences between the three analyzed EV types (Figure 5d–f and Supplementary Table S4). The size distribution of the CMDR+ particles closely corresponded to the size distribution of all particles measured in scatter mode within every EV type. After the fluorescent staining for tetraspanins and FL-NTA analysis, the size distribution of tetraspanin-positive EVs shifted for all EV types, as already previously mentioned, clearly to the left toward smaller particle sizes. In plasma-EVs, around half of all CD9 positive particles (52.17%) lie within the size range of typical exosomes between 50–100 nm. In the case of BALF- and cl-EVs, this percentage was a little lower and was around 40–45%. For all EV types, the fluorescence staining for tetraspanins exposed a fraction of very small EVs under 50 nm that was not previously visible in scatter mode and after membrane staining. In the case of plasma-EVs, this population accounted for almost 20% of all CD9+ particles. In the case of BALF- and cl-EVs, this fraction was around 10–15%. The size distributions of BALF- and cl-EVs of all particles as well as CMDR+ and tetraspanin-positive particles corresponded largely to each other. Additionally, the size distributions of CD9, CD63, and CD81 positive particles of BALF- and cl-EVs were very similar. The exact percentages of all particle fractions are listed in Supplementary Table S4.

### 3.5. FL-NTA Characterization of BALF EVs

**Figure 6:**
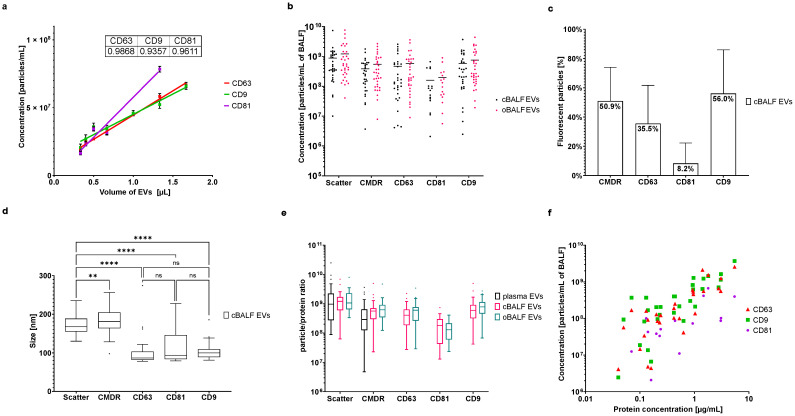
FL-NTA characterization of BALF EVs. (**a**) Concentration of BALF-derived particles measured in fluorescent mode (488 nm) after labeling with tetraspanin markers depending on the EV sample volume taken for the measurement. A line from simple linear regression was plotted and R^2^ was placed in the table for each tetraspanin marker. (**b**) Concentration of particles calculated per one mL BALF for cBALF-EVs and oBALF-EVs for all patients in FL-NTA. (**c**) The percent of fluorescent particles in comparison to all particles visible in scatter mode for cBALF EVs for all patients. (**d**) Measured mode sizes of particles in the scatter and fluorescent mode (488, 640 nm) for cBALF EVs for all patients. (**e**) Particle/protein ratio of plasma and BALF-EVs in the scatter and fluorescent mode. (**f**) Correlation between the concentration of particles and protein concentration of tetraspanin-positive cBALF-EVs. Graph (**c**) presents the mean and SD for all patients. Graphs (**d**,**e**) present the Tukey plot for all patients. **** refers to *p* value ≤ 0.0001, ** refers to *p* value ≤ 0.0021, ns refers to *p* value > 0.05 from *t*-test-paired comparison (**d**) and Wilcoxon test-paired comparison (**e**).

The fluorescent staining of BALF-EVs and their FL-NTA analysis showed linearity with particle concentrations for all analyzed tetraspanins (Figure 6a). R^2^ from linear regression for particles positive for CD63 was 0.9868, for CD9 0.9357, and for CD81 0.9611. Comparison of concentrations of particles per one mL of BALF for cBALF and oBALF showed no significant differences for all markers between these two groups (Figure 6b). No significant differences were also detected in the percent of fluorescent particles in these two groups (Supplementary Figure S5a). The representative percentages of fluorescent particles of cBALF-EVs are presented in Figure 6c. The percent of fluorescent particles in comparison to all particles visible in scatter was high in CMDR (50.9%) and CD9 (56.0%), lower in CD63 (35.5%), and the lowest in CD81 (8.2%). According to measured particle sizes, most detected differences between cBALF and oBALF were not meaningful. Only in the case of CMDR labeled particles did cBALF-EVs turn out to be slightly bigger than oBALF-EVs (Supplementary Figure S5b).

However, the particle sizes differed significantly depending on the type of fluorescent marker. The CMDR-positive EVs in cBALF EVs were detected as being meaningfully larger (mean ± SD: 183.23 ± 32.70 nm) than particles detected in scatter (mean ± SD: 171.95 ± 23.72 nm). In contrast, all the particles positive for CD63, CD9, and CD81 showed much lower sizes (mean ± SD: CD63: 100.76 ± 38.62 nm; CD9: 104.21 ± 22.11 nm; CD81: 115.81 ± 46.01 nm) than those detected in scatter (Figure 6d). The particles positive for CD63, CD81, and CD9 were all similar in size. The individual size for all measured plasma and BALF EV samples are listed in Supplementary Table S3. Based on the measured protein concentrations of the EV samples (see Supplementary Table S5), the particle/protein ratio for all EV types in scatter and after fluorescent labeling was calculated. The obtained particle/protein ratios were 100 times higher for plasma than the ratio reported by Dong et al. and 10 times higher for BALF-EVs than ratios obtained for cell culture or urine EVs [19]. Our ratios showed no significant differences between all EV types (Figure 6e). Nonetheless, there was a strong positive correlation between tetraspanin-positive particle concentration and protein concentration for BALF-EVs (Figure 6f). For cBALF, the Spearman correlation was r_s_ = 0.81, *p* < 0.0001 for CD63 EVs, r_s_ = 0.65 *p* < 0.0001 for CD9 EVs, and r_s_ = 0.81, *p* = 0.0074 for CD81 EVs. For oBALF, the Spearman correlation was r_s_ = 0.69, *p* < 0.0001 for CD63 EVs, r_s_ = 0.71 *p* < 0.0001 for CD9 EVs, and r_s_ = 0.75, *p* = 0.0007 for CD81 EVs.

### 3.6. Control-Experiments for FL-NTA

#### 3.6.1. RIPA Lysis of EVs

In order to ensure that our fluorescent staining of the EV membrane and tetraspanins identified true EVs during FL-NTA measurements, we applied differential detergent lysis [21] with RIPA of a representative standard EV sample (commercially available, lyophilized EVs derived from the NSCLC cell line, with confirmed presence of exosomal markers), expecting that fluorescent signals connected to true EVs should disappear after detergent lysis [22]. Additionally, the scatter signal should either shift left toward smaller sizes (disruption of whole EVs into smaller fragments) or decrease when the fragmentized EVs fall under the instrument’s detection limit. Our goal was to confirm that NTA properly detect EVs in our samples and our labeling methods are specific.

**Figure 7:**
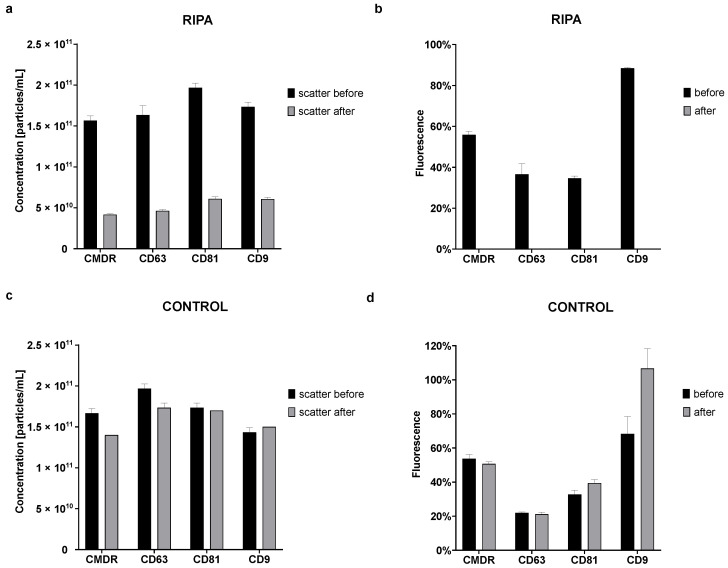
RIPA lysis of standard EVs derived from the NSCLC cell line. (**a**) The concentration of particles measured in scatter mode after labeling with CMDR and fluorescent antibodies against tetraspanin markers before and after incubation with RIPA lysis buffer. (**b**) The percent of fluorescent particles in comparison to all particles visible in scatter mode before and after incubation with RIPA lysis buffer. (**c**) The concentration of particles measured in scatter mode after labeling before and after incubation with PBS (control). (**d**) The percent of fluorescent particles in comparison to all particles visible in scatter mode before and after incubation with PBS as the control). Graphs (**a**–**d**) present the mean and SD from three replicates.

Indeed, the mean particle concentration decreased in scatter mode from 1.73 × 10^11^ ± 1.75 × 10^10^ particles/mL before treatment to 5.24 × 10^10^ ± 9.90 × 10^9^ particles/mL in RIPA treated samples. Overall, there was a 65–73% decrease in the particle concentration in scatter mode after the treatment depending on labeling type (Figure 7a). The fact that the particles were still detectable after RIPA lysis could be caused by a too low concentration of RIPA or by a too-short time of lysis. In the fluorescent mode, the decrease in detected particles was so pronounced that the number of fluorescent particles after treatment dropped down below the NTA detection limit. Before the treatment, the percent of fluorescent particles compared to all particles visible in scatter mode fluctuated depending on the marker (CMDR 55.92 ± 1.66%, CD63 36.54 ± 5.29%, CD81 34.60 ± 1.15%, and CD9 88.46 ± 0.38%), and after the treatment, there was no detectable fluorescence (Figure 7b).

In contrast, for samples treated with PBS instead of RIPA (control), there was only a slight decrease after the treatment in scatter mode (the mean concentration in scatter mode before the treatment was 1.70 × 10^11^ ± 2.19 × 10^10^ particles/mL and after the treatment 1.58 × 10^11^ ± 1.60 × 10^10^ particles/mL) (Figure 7c). Control samples also remained fluorescent after the treatment. The overall difference in fluorescence for the control samples was only 2.94% for CMDR, 0.72% for CD63, and 6.67% for CD81. For unknown reasons, the fluorescence for CD9 increased by 38.33% after the treatment (Figure 7d). The sizes of the particles in both RIPA and the control samples remained the same before and after the treatment (Figure S6).

This experiment confirmed that our labeling methods really stained exosomal markers and that we measured true EVs.

#### 3.6.2. FL-NTA Measurements of Tetraspanin-Labeled EVs after Immunomagnetic Removal of EV Subpopulations

To further prove the correctness of the performed tetraspanin-specific FL-NTA measurements of our EV samples, we investigated whether the removal of selected tetraspanin-positive EV subpopulations from the analyzed sample would be reflected by a decrease in the corresponding fluorescent signal in FL-NTA. For this experiment, we chose an oBALF-EV sample with a relatively high expression of CD63 and CD9 (we omitted CD81 due to the relatively low expression in BALF-EV samples). We removed either CD63 positive or CD9 positive EVs using magnetic beads coated with anti-CD63 or anti-CD9 antibodies, respectively. Next, we performed fluorescent labeling against CD63, CD9, and membrane labeling with CMDR.

**Figure 8:**
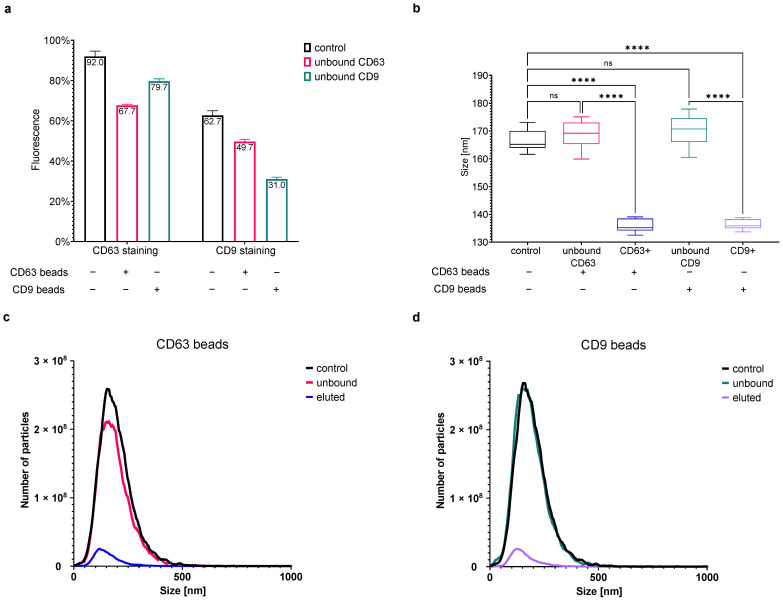
Depletion of EV-subpopulations by immune-magnetic bead separation. (**a**) The percent of fluorescent particles in comparison to all particles visible in scatter mode for oBALF-EVs of one patient. The first three bars represent the percentage CD63 positive particles, and bars 4–6 represent the percentage of CD9 positive particles relative to particles in scatter. (**b**) Measured mode sizes of particles in scatter mode of oBALF-EVs of one patient. (**c**,**d**) Distribution of number and size of detected particles in scatter mode of oBALF-EVs of one patient for the CD63 beads (**c**) and CD9 beads (**d**) experiment. For graphs (**a**–**d**), the colors stand for the following sample types: control sample (black), fraction unbound to CD63 beads (pink), fraction eluted from CD63 beads (blue), fraction unbound to CD9 beads (green), and fraction eluted from CD9 beads (violet). Graph (**a**) presents the mean and SD from three repetitions. Graph (**b**) presents the mean and SD from twelve repetitions. Graphs (**c**,**d**) present the mean from three repetitions. **** refers to *p* value ≤ 0.0001, ns refers to *p* value > 0.05, from *t*-test-paired comparison.

The fluorescent staining and subsequent FL-NTA analysis of the CD63+ EV-depleted fraction revealed a decrease in detected CD63 positive particles (in comparison to all particles measured in scatter mode). Fluorescence dropped from 92% to 68% after depletion. There was also a slight decrease in the number of CD9 positive particles from 63% to 50% (Figure 8a). Accordingly, the depletion of CD9+ EVs resulted in a higher decrease in detected CD9 positive particles and a smaller decrease in CD63 particles. The number of CD63 and CD9 positive EVs dropped to 80% and 31%, respectively. For unknown reasons, we observed an increase in the relative percentage and absolute numbers (data not shown) of CMDR positive particles.

Measurements of the negative control samples with PBS instead of EVs resulted in no signal in both scatter and fluorescence mode (data not shown). We also eluted the bead-bound EVs using a commercial elution buffer and stained them accordingly with CMDR and tetraspanins. We could measure the eluted beads in scatter mode, whereas the measurements in fluorescence mode detected no or only low and not reproducible percentages of fluorescence-positive particles (both for tetraspanins and CMDR—data not shown). This could be due to several reasons. First, the relatively low number of captured EVs imposed a low end-dilution of the sample for the NTA-measurement. It caused a higher than usual dilution of the fluorescent antibodies or CMDR at the time of staining, which could decrease the staining efficiency. Additionally, we suspected that the used elution buffer negatively impacts the CMDR and tetraspanin staining since we observed a decrease in the fluorescent signals after EV staining in the presence of the elution buffer only (data not shown).

Interestingly, comparing the size of the particles detected in scatter mode of the bead depleted EV-fractions with the corresponding CD63+ or CD9+ eluted EV-fractions, we once again obtained a confirmation of the smaller size of tetraspanin-positive EVs. The mode size of the particles remaining after bead depletions measured in scatter mode was only slightly larger than the control (168.9 ± 4.6 nm for the CD63 unbound and 170.2 ± 5.0 nm for the CD9 unbound population vs. 166.8 ± 3.7 nm for the control). The captured EVs’ mode size was significantly smaller (135.8 ± 2.3 nm for the eluted CD63+ and 136.4 ± 1.8 nm for the eluted CD9+ population; see Figure 8b). The determination of the captured EVs’ mode size in fluorescent mode was hindered by the low particle number and therefore not statistically assured for all measured samples. However, the measured mode sizes between 80–120 nm in single samples of the captured CD63+ and CD9+ EVs (data not shown) corresponded to the size of previously detected tetraspanin-positive particles in whole EV preparations, as described above.

Admittedly, the number of captured EVs was relatively low, looking at the measured concentrations in scatter (Figure 8c,d) and the still high percentage of tetraspanin-positive particles in the unbound-fraction. Obviously, further optimization of the method would be required to obtain better results. A longer incubation time or different bead–EV ratio could increase the captured EV number. However, since this experiment was intended only as a proof-of-concept and was not designed to provide exact values, we did not aim for complete removal of all CD63 or CD9 positive EVs or exactly checked the efficiency of EV removal. In summary, this proof-of-concept experiment verified our FL-NTA immunolabeling technique for the detection of tetraspanin-positive EVs.

#### 3.6.3. Impact of Plasma Lipoproteins on FL-NTA Measurements

**Figure 9:**
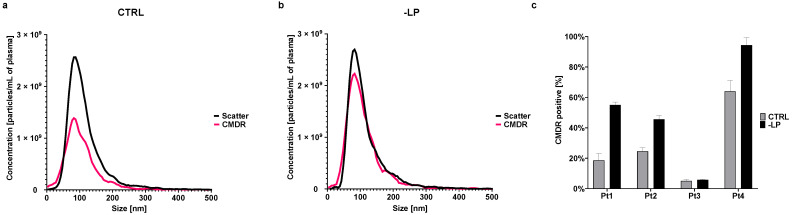
Lipoprotein removal from plasma EVs. (**a**) Distribution of particles in the scatter and fluorescent mode (640 nm) for plasma EVs of a representative patient during NTA measurement (CTRL). (**b**) Distribution of particles in the scatter and fluorescent mode (640 nm) for plasma EVs of a representative patient during NTA measurement after lipoprotein removal (-LP). (**c**) The percent of CMDR positive particles in comparison to all particles visible in the scatter mode of plasma EVs before and after lipoprotein removal. Graphs (**a**,**b**) present the mean from four replicates. Graph (**c**) presents the mean and SD from three (Pt1) to six (Pt2–4) replicates.

To verify our assumption that the high lipoprotein content in our plasma EV-isolates interferes with CMDR labeling and prevents the detection of tetraspanin-positive EVs in FL-NTA, we removed lipoproteins from plasma prior to EV isolation. After lipoprotein removal (-LP) and EV isolation by SEC, we did not observe a change in total particle concentration or size of the particles in scatter mode compared to the control (CTRL), where there was no lipoprotein removal step before the isolation. Mean concentration in -LP was 4.10 × 10^10^ ± 2.19 × 10^9^ particles/mL and in CTRL it was 4.08 × 10^10^ ± 3.40 × 10^9^ particles/mL. However, we observed a significant increase in CMDR-positive particle concentration (Figure 9a,b). The mean concentration of CMDR+ particles in -LP was 3.83 × 10^10^ ± 2.50 × 10^9^ particles/mL and in CTRL 2.58 × 10^10^ ± 2.77 × 10^9^ particles/mL. Measurement of the content of CMDR+ particles by FL-NTA of EVs separated from plasma of four different patients after removing lipoproteins showed a significant increase in CMDR positive particles in comparison to the control samples in three patients (Figure 9c). In the case of the staining for tetraspanins, we unfortunately obtained non-reproducible results (data not shown). For the plasma-EV samples for which no tetraspanins could previously be detected by NTA, lipoprotein removal did not improve the detection. For other EV samples where CD9 positive EVs could be initially detected, the percentage of CD9+ EVs increased in the -LP sample. However, this result was not explicitly reproducible for all tested samples.

### 3.7. Correlation of BALF or Plasma-EVs Characteristics with NSCLC Patient Diagnosis

Given that within our small patient cohort, six patients during the diagnosis process turned out to have a lung lesion other than NSCLC, and in three patients the lung tumor could not be unequivocally confirmed, we decided to look at the potential of any of the investigated EV-related markers to differentiate between NSCLC patients and patients with other lung lesions. However, for none of the investigated parameters of both plasma and BALF-EVs (total particle number, particle size, CMDR+ particle number, tetraspanin-positive EV number, etc.), was a significant difference observed. We also could not find a correlation of the investigated EV-metrics with any of the clinical parameters (data not shown). One reason could be the small size of the patient cohort, which did not allow statistical significance to be reached. Another reason is that plasma and BALF-derived EVs from patients contain both cancer-derived as well as normal EVs, so that general EV-markers may not be powerful enough to diagnose NSCLC patients.

## 4. Discussion

### 4.1. Selection of Isolation Methods for Plasma- and BALF-EVs

In the last several years, EVs have emerged as a promising new version of a liquid biopsy in cancer treatment. Playing a fundamental role in cell communication within the tumor microenvironment and mediating immunoinhibitory and pro-tumorigenic signals, they are under intensive research as potential biomarkers in diagnosis, prognosis, and treatment response or as therapeutic drug carriers.

It seems that plasma or serum-derived EVs are the easiest accessible sources of EV-based biomarkers. Unfortunately, their molecular characterization and translation into the clinic have been impeded by challenges to isolate EVs with sufficient yield and purity. This is because plasma contains a high concentration of proteins (mostly albumin, 35–55 mg/mL) and several orders of magnitude more lipoproteins (~10^16^/mL) than EVs (~10^7^–10^9^/mL) [23]. Several methods have been described for the separation of EVs. which vary in purity and yield of the received EV isolate. SEC is the most common method for EV enrichment from plasma since it removes the most contaminating proteins and allows for the purification of EV-enriched fractions from LDLs and HDLs. The method results in relatively pure and intact EVs, are broadly described in the literature, and are already well established in our laboratory, in contrast to differential ultracentrifugation (UC), which is not recommended for isolating EVs from plasma [24,25]. Therefore, we used centrifugation and homemade SEC columns for plasma EVs.

Another promising source of tumor derived EVs in lung cancer patients seems to be BALF. Unfortunately, BALF and plasma have a completely different composition and volume, and therefore each requires a unique approach for EV separation. Because BALF does not contain lipoproteins or high amounts of protein, we did not expect high contaminations. At the time of method development, there was no available SEC method for large volumes of fluid and our attempts to concentrate it were unsuccessful because of its high viscosity. Since the literature suggests UC as a good isolation method for BALF, we decided to use it [26]. During method optimization, we also performed an additional SEC purification step of the resuspended BALF-EV pellet after UC and compared it to EVs isolated only by UC. We found no differences in the EV profile measured by NTA (size), but noticed a substantial drop in EV recovery, which would leave not enough material for all planned analyses (data not shown). For these reasons, we decided to omit this step.

### 4.2. Characterization of EVs in the Context of Standardization and Previous Reports

The importance of standardization and parameter monitoring was strongly emphasized by Vestad et al. [27]. Even small changes can lead to different measured concentrations and sizes of particles. Mørk et al. [28] noticed in their paper the loss of quality when one analyzed EV-enriched particle fractions after a freeze–thaw cycle. We performed our NTA experiments on freshly isolated EV preparations from frozen plasma. However, in the case of BALF, NTA experiments were performed on previously isolated and frozen EV samples for technical reasons. However, our cryo-TEM pictures of thawed EVs from BALF proved that the phospholipid bilayer remained intact, and the EVs kept their usual shape and integrity (Figure S7a).

Using MISEV-recommended “classical” EV characterization methods, we showed that our EV separation methods were effective. We managed to detect classical transmembrane (CD9, CD81) and cytosolic (Tsg101, syntenin) EV-markers in both plasma and BAL-EVs and excluded secretory pathway contaminants (calnexin). Our NTA analysis in the scatter mode of both EV types suggested that plasma may be a better source of EVs in lung cancer patients since in comparison to BALF, it contained approximately 500 to 250 times more particles per mL of more homogenous size and around 3.5 times more exosomal proteins. Rodriguez et al. [29] similarly noticed higher particle numbers in BALF than in plasma, however, there, the obtained particle number was 5–10 times lower in BALF and 1000 times lower in plasma compared to our results. This could be explained by the different isolation methods and pooled samples used by Rodriguez. In contrast, we performed all of our analyses pairwise, comparing plasma, oBALF, and cBALF EVs separately for every patient. The lower EV numbers in BALF were expected since BALF is not a “true” biological fluid, but is obtained by diluting some original biological material with saline solution and contains much fewer EV donor-cells.

### 4.3. Membrane Labeling Reveals EV Sample Purity

Our further NTA-based identification of “true” EVs based on CMDR membrane staining and tetraspanin detection showed that a substantial proportion of the particles measured by NTA in scatter mode, especially in the case of plasma, were of non-vesicular origin and rather represented protein aggregates and lipoproteins than EVs. Membrane labeling revealed that BALF contained a higher proportion of true EVs than plasma, with around 50% of CMDR-positive particles in BALF in comparison to only 30% in plasma. Indeed, our cryo-TEM analysis already showed significantly higher contamination of the plasma-derived EV-sample with protein aggregates and single-membrane vesicles in comparison to the BALF sample; although rare per field, only double-membrane bona-fide EVs were visible.

Additionally, we noticed that the EV mode size increased after labeling with CMDR by around 10–20 nm in all of our EV sample types, which is in line with the observations after EV labeling with PKH [30] or the FM dye [31]. An explanation for this observation may be the intercalation of CMDR molecules into the EV membrane, causing an increase in size. In addition, particularly smaller particles probably representing protein aggregates that are not stained with CMDR, contribute to a smaller mode size in scatter mode and are eliminated in the fluorescence measurement. Furthermore, our preliminary experiments optimizing the dye concentrations showed a strong particle size increase at very high CMDR concentrations. This points to the possibility of the aggregation of CMDR particles that are then detected by the NTA, causing a shift in the particle size distribution toward bigger sizes, as also observed by Wu Y et al. [32].

### 4.4. Lipoprotein Influence on EV-Membrane Labeling and NTA-Analysis

The results discussed above point to the possibility that our SEC method does not provide a full separation of plasma EVs from lipoproteins due to the overlap in size, which was already previously observed [33,34,35]. The lipoproteins in plasma are composed of very-low-density lipoproteins (VLDLs), intermediate and low-density lipoproteins (IDLs and LDLs), high-density lipoproteins (HDLs), and chylomicrons, which all interfere with the characterization of EV preparations from this source including NTA measurements [23]. Measuring particles directly in plasma by NTA, Gardiner et al. shown that lipoproteins may account for more than 98% of particles [10].

SEC-isolation of EV fractions removes contaminating HDLs and LDLs due to their small size below 30 nm. However, bigger VLDLs and chylomicrons may still be present, as evident by their triglyceride or Apo-B content [36]. To address this issue, we performed additional western blot analysis of our EV isolates for the presence of lipoprotein marker Apo-B. We managed to detect it in our plasma EV samples, but not in the BALF EVs or cell lysate (Supplementary Figure S8).

Although staining with membrane dyes may help exclude some impurities mimicking EVs from analysis, it will not fully differentiate between true EVs and lipoproteins or even protein aggregates. Recently, it has been discovered that fluorescent lipophilic membrane dyes such as PKHs, DiD, or Cell Mask dyes, which are commonly used to identify true EVs, are not specific to the vesicular membrane and can be incorporated into any lipid structure including lipoproteins and also bind to free proteins [37]. Takov et al. showed that fluorescent dye transfers to the target cell after staining of the small EVs (sEVs) obtained from plasma by SEC did not correlate with sEV content. The authors observed a similar or even higher fluorescent uptake of vesicle-poor but protein-rich SEC fractions. They concluded that lipoproteins and free proteins unavoidably co-isolated with sEVs significantly contribute to the fluorescent membrane dye’s transfer and uptake by target cells [35].

So far, no studies have investigated the impact of lipoproteins on NTA measurements after membrane dye staining. In our case, we observed a lower percentage of CMDR-positive particles in plasma-EV samples in comparison to the BALF-EV samples. We suspected that the lipoproteins present in our plasma EV samples interfered here with the staining and NTA-measurement. They may compete with true EVs for dye binding, interfere with EV labeling, and contribute in an undefined way to the CMDR+ particle count detected by NTA. Furthermore, some of the remaining LDLs and HDLs not removed by SEC could also incorporate the dye, reducing the available dye amount for EV staining. However, due to their small size, they would be under the ZetaView instrument’s detection limit. Based on the previous reports and our results, we concluded that lipophilic dyes might not be reliable for labeling small EVs from plasma unless an entirely pure population without proteins and lipoproteins of EVs is obtained, which given the currently available isolation methods has not been achieved thus far.

Attempts to increase the purity of EV preparations from plasma by including additional gradient separation or differential UC have only been partially successful [38]. This also has some implications for sample collection since EV-enriched plasma samples collected in the postprandial state demonstrated an increase in total particle numbers in NTA [28,39,40,41]. On one hand, which exposes the possible strong interference of lipoproteins with NTA-measurements and, on the other hand, the need to analyze plasma EV samples in the fasting state. In our case, food intake before blood draw was not a strict exclusion criterion in our patients’ group, however, most of the blood samples were routinely drawn in the fasting state. The strong interference of lipoproteins with NTA-measurements also implies that direct measurements of EVs in unpurified plasma by NTA in scatter mode, even though it is technically possible and has already been performed by several researchers [10,18,22], may lead to a high overestimation of the actual EV numbers and have to be interpreted with caution. To further complicate the issue, recently, an in vitro association of LDLs with EVs and their interference with vesicle analysis has been observed [22].

### 4.5. Antibody Labeling Show Significant Differences between Plasma and BALF EVs

Given the CMDR-labeling inaccuracy in EV number evaluation, we performed staining of our EV samples with fluorescent antibodies directed against tetraspanins as classical small EV or exosomes markers. We detected expression of all tested tetraspanins on BALF-derived EVs, with the amount of detected CD9-positive particles being the highest and corresponding to the amount of detected CMDR+ vesicles and the amount of CD81 positive particles being the lowest. Interestingly, all tetraspanin-labeled samples detected in fluorescent mode showed a decreased mean size in comparison to the whole vesicle population in scatter mode or the CMDR+ population. On one hand, this was expected since tetraspanins are regarded to be predominantly markers of smaller EVs of endosomal origin, named exosomes. It seems that in our BALF-EV population, preferably the smaller exosomal EVs around 100 nm express tetraspanins, and their presence is becoming less common the larger the EVs. On the other hand, we were surprised to note that the detected tetraspanin-positive particle size distribution curve did not fully overlap with the scatter or the CMDR+ distribution curves and shifted toward smaller sizes. Understandably, the smallest tetraspanin-positive EVs were not detected in the scatter mode or after CMDR staining. One explanation for this phenomenon could be the technical limitation of the NTA instrument. In highly polydispersed samples such as EV preparations from biological fluids, large and therefore strongly light-scattering particles overshadow the smaller particles and exhibit halo effects in scatter mode. Additionally, in comparison to polystyrene beads, EVs have a very low refractive index. Thus, they may lie under the ZetaView instrument’s detection limit in scatter mode and may also be too small to incorporate enough CMDR dye to be detected in the fluorescent mode. After labeling with tetraspanin-specific fluorescent antibodies, bigger tetraspanin-negative particles become invisible, whereas the smaller tetraspanin-positive EVs become traceable in the fluorescent mode. Similar observations were made by other researchers. Oesterreicher et al. detected higher EV-specific CD63 and CD81 marker expression in the small vesicle range (<200 nm) than in the intermediate and large ranges [42]. Staining with quantum dots for CD9 of canine MSC-derived EVs resulted in the detection of small particles ranging in size from 30–100 nm, which were significantly less numerous in scatter mode [43]. The authors concluded that these smaller fluorescent particles could be CD9 positive small EVs not visible in scatter or free quantum dots and quantum dots aggregates. Here, by evaluating the fluorescence background of the control samples (fluorescent antibodies only) and adjusting the instrument settings accordingly, we could largely exclude the detection of free antibodies or antibody aggregates. In our study, the peak shift in fluorescent mode can be attributed to tetraspanin-bearing small EVs not detectable in scatter mode (which also explains the over 100% scatter/fluorescent mode particle ratios of some samples). Our control NTA-measurement of CD63 and CD9 labeled BALF-EVs after EV-depletion by CD63- or CD9-specific magnetic beads confirmed that we detected true CD63 or CD9 positive EVs in fluorescence NTA since EV-depletion markedly reduced the tetraspanin-specific fluorescence.

In contrast, we could not detect any tetraspanins in our plasma EV samples at the same staining conditions except only single samples. We believe that the reason lies in the high concentration of contaminating lipoproteins, which do not express tetraspanins, but are detected in the scatter mode of the instrument. Since we have to adjust the dilution of the sample for measurement based on the particle count in scatter mode, the lipoprotein contamination of the plasma-EVs impose a high sample dilution factor to stay within the optimal measurement range of the instrument. This dilution is then too high to detect the low abundant tetraspanin-positive EVs in fluorescent mode. However, biological reasons may also be possible (e.g., plasma EVs may have less tetraspanin-epitopes on their EV surface than BALF-EVs and may therefore be less detected by the instrument. Indeed, direct phenotyping of plasma-derived EVs by nano flow cytometry revealed a very low percentage of tetraspanin-positive EVs, which barely exceeded 4% [19]. Furthermore, only ultracentrifugation and SEC with ultrafiltration as EV-isolation methods resulted in detectable numbers of tetraspanin-positive EVs. In contrast, EVs from the cell culture supernatant or urine showed significantly higher tetraspanin expression rates between 25–40%, which is comparable to our results obtained from BALF. An increase in sample input for the measurement to reach the detection level is not feasible for NTA since a too concentrated sample will fall out of the instrument’s linear range.

Sodar et al. showed that even after applying the most efficient and purifying EV isolation methods currently available, the obtained EV samples still contained at least one order of magnitude less “true” EVs in comparison to the contaminating lipoproteins and protein aggregates [22]. Therefore, we decided to remove lipoproteins from the plasma sample prior to our standard EV isolation using a commercially available kit. We observed an increase in the % of detectable CMDR-positive particles in most cases, indicating that the presence of lipoproteins in the EV isolates indeed interfered somehow with EV membrane staining. Unfortunately, the staining against tetraspanins after lipoprotein removal provided somewhat contradictory results (in some samples an increase, in others no change—data not shown). Right now, we do not know exactly what the mechanism of lipoprotein interference during EV labeling and FL-NTA detection is, but our experiment confirms that it has an impact on the results. Mork et al. performed a similar experiment, but the NTA-measurements were performed directly in platelet-free plasma (PFP) without EV-isolation and only in scatter mode. Lipoprotein removal resulted in a median reduction of 62% of the measured particle concentration, once again emphasizing the fact that scatter NTA-measurements do not only detect true EVs [18].

### 4.6. NSCLC Patients Differentiation

None of the investigated general EV-parameters could differentiate between NSCLC patients and non-NSCLC patients or correlated to any clinical parameter. Although several studies have already shown a correlation between total EV levels in plasma and disease activity and progression [44], recently the EV research has moved toward the investigation of more specific EV-cargo as a diagnostic or prognostic marker (e.g., the presence of immunosuppressive factors, cancer-specific molecules or miRNAs). In our ongoing studies, we plan to further characterize the molecular cargo of BALF-EVs in the context of EV-mediated immunosuppression in the lung TME in a much larger cohort of NSCLC patients. We are convinced that a comprehensive examination of the molecular composition of BALF-EVs might provide specific EV-cargo signatures that will be more accurate and reliable diagnostic or prognostic biomarkers than any single soluble BALF or plasma biomarker.

### 4.7. Conclusions

**Figure 10:**
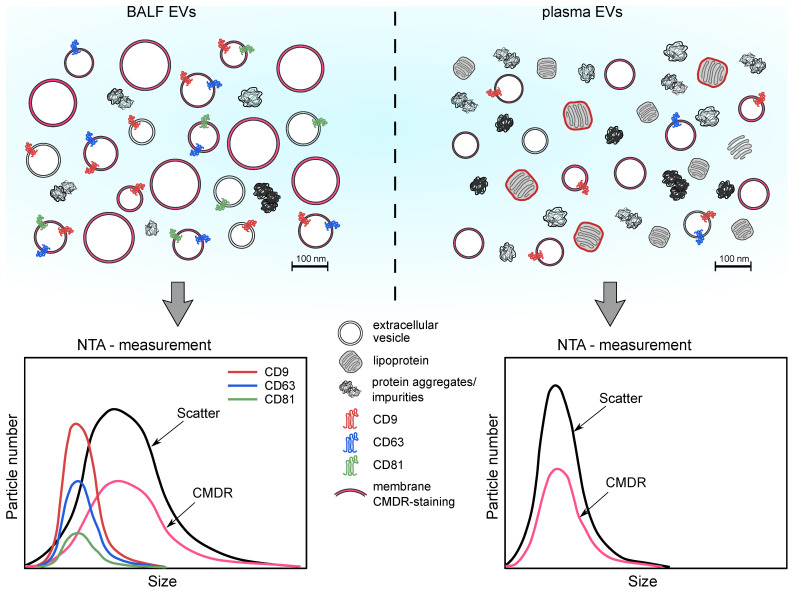
BALF and plasma EV characterization summary.

In summary, in this study, we presented the first comprehensive phenotyping of BALF-derived EVs from lung cancer patients using classical EV-characterization methods as well as the relatively new FL-NTA method. In addition, we have shown that EV immunolabeling for specific EV markers combined with the differential use of the scatter mode and fluorescent mode NTA analysis can provide the concentration, size, distribution, and surface phenotype of bona fide EVs in a heterogeneous solution. By performing FL-NTA analysis of BALF-derived EVs in comparison to plasma-derived EVs, we have revealed that this method is suitable only for relatively pure EV isolates such as BALF or CCM. In particular, EV preparations from plasma or serum, with very low EV levels in comparison to contaminating lipoproteins, are less suitable for FL-NTA phenotyping, and even membrane-specific labeling might strongly overestimate EV numbers. The different composition of BALF-EV versus plasma EV samples and its impact on NTA analysis are summarized in Figure 10. Development of applicable purification methods for these EV preparations to remove lipoproteins, as has recently been attempted by Onodi et al. [45], and further refinement of the immunolabeling process and optimization of the FL-NTA settings are needed for the analysis of such polydispersed EV preparations. Further development of FL-NTA based EV-phenotyping toward the detection of more specific cargo such as cancer-biomarkers will advance our understanding of the composition and quality of different EV preparations. This is indispensable before a conclusive statement about their biological function and clinical significance can be made.

## Data Availability

Additional supporting information may be found online in the Supplementary Materials or are available from the corresponding author upon reasonable request.

## Supplementary Materials

The following are available online at https://www.mdpi.com/article/10.3390/cells10123473/s1, Supplementary Table S1: Fluorescent label, antibodies, isotype controls, and secondary antibody used for NTA, flow cytometry, and western blot analysis; Supplementary Table S2: Concentration of particles for 1 mL of plasma/BALF in scatter, membrane, and tetraspanin labeling; Supplementary Table S3: Size of particles for 1 mL of plasma/BALF in scatter, membrane, and tetraspanin labeling; Supplementary Table S4: The concentration of particles—the fraction of all particles [%] in six size fractions (<50 nm, 50–100 nm, 100–150 nm, 150–200 nm, 200–250 nm, >250 nm) for plasma, BALF, and cell line EVs; Supplementary Table S5: Concentration of protein for 1 mL of plasma/BALF; Figure S1: EVs separation from plasma on SEC—particle/protein ratio; Figure S2: Flow cytometry analysis of oBALF-EVs bound to magnetic Dynabeads coated with antibodies against tetraspanins; Figure S3: Variability of the measured size and concentration of standard beads on NTA; Figure S4: Optimization of CMDR concentration; Figure S5: Antibody labeling of cBALF and oBALF EVs; Figure S6: RIPA lysis of EVs—size; Figure S7: Cryo-TEM images of thawed cBALF EVs; full blots from Figure S2a; Figure S8: Lipoprotein marker Apo-B; raw pictures from Cryo-TEM from Figure S2b.
