# Supplementary material for: Characterization of Extracellular Vesicles from Bronchoalveolar Lavage Fluid and Plasma of Patients with Lung Lesions Using Fluorescence Nanoparticle Tracking Analysis
Source: Cells. 2021 Dec 9;10(12):3473. doi: 10.3390/cells10123473 (PMC8699990; doi:10.3390/cells10123473)

Supplementary data:

Supplementary Table S1. Fluorescent label, antibodies, isotype controls, and secondary antibody used for NTA, flow cytometry, and Western blot analysis

Antibody/dye	Source, Catalog number:	Antigen	Dilution	Application
CellMask™ Deep Red plasma membrane stain	Invitrogen™ Thermo Fisher Scientific, C10046	Membrane Stain	1:1250000	NTA
PE	Invitrogen™ Thermo Fisher Scientific, MA1-19650	CD63	1:5000	NTA
PE	Biolegend, 349506	CD81	1:40000	NTA
PE	Biolegend, 312106	CD9	1:6000	NTA
FITC	Beckman Coulter, IM1755U	CD9	1:20	Flow cytometry
PE	BD Pharmingen™, 556020	CD63	1:20	Flow cytometry
APC	Beckman Coulter, A87789	CD81	1:20	Flow cytometry
Mouse IgG1 κ Isotype Control -PE	eBioscience™, 12-4714-82	Isotype Control PE	1:10	Flow cytometry
Mouse IgG2b κ Isotype Control -APC	eBioscience™, 17-4732-81	Isotype Control APC	1:10	Flow cytometry
Mouse IgG2b κ Isotype Control-FITC	eBioscience™, 11-4732-81	Isotype Control FITC	1:10	Flow cytometry
Anti-ALIX	abcam, ab117600	ALIX	1:1000	WB
Anti-Calnexin	abcam, ab13504	Calnexin	1:2000	WB
Anti-CD9	Invitrogen™ Thermo Fisher Scientific, 10626D	CD9	1:500	WB
Anti-CD81	Invitrogen™ Thermo Fisher Scientific, 10630D	CD81	1:1000	WB
Anti-NY-ESO-1	Santa Cruz Biotechnology, Sc-53869	NY-ESO-1	1:200	WB
Anti-Synthenin	abcam, ab19903	Synthenin	1:1000	WB
Anti-Tsg-101	abcam, ab30871	TSG-101	1:1000	WB
Biotin Anti-Alipoprotein B	Abcam, ab20898	Apo-B	1:1000	WB
HRP	Thermo Fisher Scientific, A16078	mouse antibodies	1:10000	WB
HRP	Thermo Fisher Scientific, A16110	rabbit antibodies	1:10000	WB
HRP	Santa Cruz Biotechnology, Sc-2020	donkey anti-goat antibodies	1:10000	WB

Supplementary Table S2. Concentration of particles for 1 mL of plasma/BALF in scatter, membrane, and tetraspanin labeling

	Plasma EVs			cBALF EVs					oBALF EVs				
Pt	Scatter [particles x10^{-7}/mL plasma]	CMDR [particles x10^{-7}/mL plasma]	CD9 [particles x10^{-7}/mL plasma]	Scatter [particles x10^{-7}/mL BALF]	CMDR [particles x10^{-7}/mL BALF]	CD63 [particles x10^{-7}/mL BALF]	CD81 [particles x10^{-7}/mL BALF]	CD9 [particles x10^{-7}/mL BALF]	Scatter [particles x10^{-7}/mL BALF]	CMDR [particles x10^{-7}/mL BALF]	CD63 [particles x10^{-7}/mL BALF]	CD81 [particles x10^{-7}/mL BALF]	CD9 [particles x10^{-7}/mL BALF]
1	6600.0	2333.3	2533.3	136.0	86.0	55.7	41.6	123.0	101.3	27.2	80.8	28.7	147.7
2	4733.3	1966.7		68.0	17.1	10.3		39.7	29.0	7.3	6.9		10.6
3	3466.7	2600.0		1.0	0.4	0.4	0.2	0.7	40.0	14.7	64.1	5.5	48.0
4	41866.7	6080.0		4.5	2.5	0.4		0.2	143.6	92.8	54.2	17.6	69.1
5	6666.7	1873.3		153.3	33.7	13.7		21.1	97.5	68.1	36.0	13.2	20.8
6	24000.0	6933.3		91.8	56.5	25.1	8.6	40.0	55.6	37.1	80.4	5.2	22.8
7	22533.3	7866.7		5.0	2.8	0.5		1.4	42.6	19.3	10.7		26.2
8	1033.3	56.0		34.7	15.1	7.7	3.3	12.6	56.7	29.3	8.5		74.7
9	4533.3	1493.3		106.7	62.1	45.7		127.8	12.5	5.0	2.0		7.3
10	9066.7	4466.7	3600.0	52.8	62.4	66.4		70.4	225.3	269.3	69.3		110.7
11	8000.0	3166.7		96.7	59.3	54.0	44.0	62.7	125.2	61.3	5.3	4.4	12.3
12	28666.7	2000.0		118.8	58.6	212.7		63.7	47.6	22.3	15.1		13.5
13	122000.0	24666.7							4.1	0.8	0.9		1.9
14	26796.0	9034.7		23.6	11.9	16.0	10.0	37.0	24.7	18.6	16.9	9.5	27.1
15	6066.7	3866.7		19.2	9.2	16.8	1.3	36.7	145.7	81.4	82.9	6.6	138.6
16	26400.0	7333.3		114.2	75.8	153.8	66.7	143.6	35.4	18.2	19.1		21.4
17	32266.7	9466.7		35.3	14.4	19.1		14.3	50.0	28.5	44.0		37.0
18	8133.3	2160.0		16.7	7.1	4.1		8.5	36.0	17.5	11.9		16.8
19	28533.3	2733.3							19.9	11.6	25.4		14.6
20	2113.3	333.3		147.0	39.5	125.0	10.2	163.0	34.7	9.5	19.0		20.1
21	4466.7	2533.3		33.0	4.7	10.0	1.1	21.4	26.0	7.3	10.9	1.2	26.7
22	1980.0	613.3		14.1	5.4	1.5		1.9	566.0	216.0	159.2	90.0	238.0
23	6800.0	546.7		122.0	25.1	56.0	8.6	115.0	11.3	3.5	2.5	0.6	17.1
24	34000.0	15066.7		15.0	7.0	3.4		8.2	15.0	9.0	4.3		7.7
25	10666.7	3333.3		31.1	22.6	9.1	4.2	16.7	76.0	54.9	20.7	2.9	58.1
26	16200.0	7466.7		35.7	12.7	8.5		9.6	233.3	96.0	156.5	23.0	255.4
27	2133.3	810.0		148.5	54.1	60.3	7.3	82.0	267.8	89.3	110.0	30.4	130.7
28	1960.0	600.0		34.0	32.0	3.8			15.9	9.8			
29	12533.3	5866.7		46.7	44.0			30.0	80.7	36.7			60.0
30	39600.0	13333.3		198.0	137.0	178.0		159.0	265.0	172.0	285.0		229.0
31	260000.0	30666.7		739.2	177.8	257.0	39.6	369.6	766.7	142.0	360.0	65.3	440.0
32	2573.9			39.1	17.2	12.9	3.8	20.4	12.9	7.4	2.9		3.2
33	8800.0	3800.0	540.0	111.2	50.0	12.2	5.1	20.5	413.3	122.0	82.0	16.0	174.7
34	15466.7	1566.7		37.9	27.1	5.6		9.3	84.3	49.5	16.0		21.7

Supplementary Table S3. Size of particles for 1 mL of plasma/BALF in scatter, membrane, and tetraspanin labeling

Pt.	Plasma EVs			cBALF EVs					oBALF EVs				
	Scatter [nm]	CMDR [nm]	CD9 [nm]	Scatter [nm]	CMDR [nm]	CD63 [nm]	CD81 [nm]	CD9 [nm]	Scatter [nm]	CMDR [nm]	CD63 [nm]	CD81 [nm]	CD9 [nm]
1	94.6	105.9	88.6	195.8	193.9	80.9	79.6	93.2	197.3	201.2	78.9	84.5	89.7
2	102.7	113.2		167.0	181.1	83.5		105.1	153.7	171.0	95.7		95.7
3	99.6	98.4		217.9	243.0	274.2	227.5	185.6	151.4	177.5	78.0	84.8	81.5
4	109.2	154.5		147.4	154.0	105.2		125.5	161.4	170.4	85.5	176.0	97.1
5	83.4	102.0		140.0	128.2	84.3		102.1	167.7	178.9	107.6	160.0	125.3
6	98.9	123.8		159.6	161.6	104.4	79.2	103.0	151.1	152.1	88.8	78.4	92.9
7	104.4	117.5		179.0	162.8	161.5		138.0	179.5	206.6	97.7		108.3
8	91.4	117.3		152.7	164.8	92.9	96.0	100.6	150.4	160.2	87.0		84.0
9	112.4	129.0		196.3	192.0	103.0		100.1	158.8	165.4	94.1		113.3
10	102.9	106.0	77.8	196.3	192.0	103.0		100.1	170.5	164.2	78.6		85.4
11	98.4	108.0		154.1	161.3	79.0	89.4	90.6	161.0	164.7	83.2	176.2	86.9
12	89.5	128.5		166.2	175.3	81.9		96.8	164.3	169.8	92.9		111.8
13	95.3	114.4							173.6	169.7	131.2		120.0
14	119.1	132.1		191.6	208.4	80.2	196.1	84.0	189.0	195.1	85.5	79.0	91.5
15	104.3	110.6		158.3	169.8	102.6	109.4	90.2	166.2	175.6	112.0	92.7	98.3
16	109.2	131.3		163.6	174.9	80.0	92.3	81.5	156.7	162.4	76.1		85.8
17	107.5	118.9		185.1	188.0	86.7		102.1	173.4	172.1	80.7		92.7
18	106.1	126.5		211.2	256.5	84.3		88.1	189.0	182.6	86.6		106.2
19	112.8	154.8							169.3	179.9	76.3		82.0
20	95.0	122.1		169.0	209.1	79.3	87.8	87.4	161.8	198.7	78.6		82.0
21	83.7	88.9		177.2	214.7	84.9	117.8	89.0	171.8	192.9	93.6	152.4	90.6
22	93.9	110.7		163.5	188.8	97.1		140.9	160.1	159.9	89.4	157.2	98.3
23	102.2	140.6		150.2	218.9	78.4	93.7	81.5	179.4	217.6	83.7	91.3	88.4
24	89.4	98.1		185.9	218.9	121.5		109.5	173.4	196.0	97.1		92.1
25	84.3	95.6		153.6	156.6	82.7	159.1	97.9	150.8	157.7	80.8	205.9	80.6
26	83.8	88.9		157.5	169.7	87.0		109.3	147.3	155.8	78.8	222.9	87.0
27	89.6	101.8		159.3	167.9	82.6	83.7	97.3	162.5	178.4	89.5	170.6	91.3
28	95.5	119.1		235.1	242.8	166.5			186.3	184.9			
29	107.3	117.0		129.8	97.8			82.4	181.7	173.4			79.4
30	107.6	128.3		189.2	184.8	91.2		122.0	184.2	191.5	90.0		103.3
31	113.7	158.5		168.0	170.8	84.2		98.8	148.1	149.0	89.8	93.4	89.4
32	84.5			139.1	151.6	78.0	79.8	80.9	144.3	147.9	82.7		107.8
33	85.4	93.9	103.5	172.4	181.3	105.8	145.8	126.5	153.4	166.2	94.7	107.6	102.2
34	89.1	115.3		170.6	182.0	96.9		120.6	174.9	187.8	103.7		127.9

Supplementary Table S4. The concentration of particles – the fraction of all particles [%] in six size fractions (< 50nm, 50–100 nm, 100–150 nm, 150–200 nm, 200–250 nm, >250 nm) for plasma, BALF, and cell line EVs

	plasma EVs			oBALF EVs					NSCLC Cell line EVs				
	Scatter	CD9	CMDR	Scatter	CD63	CD9	CD81	CMDR	Scatter	CD63	CD9	CD81	CMDR
[nm]	[%]	[%]	[%]	[%]	[%]	[%]	[%]	[%]	[%]	[%]	[%]	[%]	[%]
< 50	1.44	19.59	2.52	0.42	8.42	7.67	13.96	0.56	0.62	12.88	16.69	9.75	1.09
50–100	42.54	52.17	37.18	7.11	43.28	37.21	39.52	5.83	15.18	44.02	46.96	39.90	19.77
100–150	38.44	26.00	39.74	24.04	24.94	27.18	23.26	20.84	39.07	20.55	23.75	30.25	36.18
150–200	11.97	1.12	13.10	25.37	12.21	15.12	9.30	24.40	25.80	11.73	7.70	12.04	23.14
200–250	3.47	0.00	4.42	18.54	5.78	6.48	4.65	18.35	11.04	6.95	3.00	3.62	10.76
> 250	2.14	1.12	3.03	24.53	5.37	6.34	9.30	30.02	8.28	3.85	1.90	4.43	9.06

Supplementary Table S5. Concentration of protein for 1 mL of plasma/BALF

Patient no.	The concentration of protein in plasma EVs [ug/mL of plasma]	The concentration of protein in cBALF EVs [ug/mL of BALF]	The concentration of protein in oBALF EVs [ug/mL of BALF]
1	31.99	1.47	2.21
2	121.86	0.42	0.08
3	149.72	0.16	1.03
4	16.71	0.04	1.37
5	248.20	1.03	0.52
6	130.64	0.44	0.23
7	306.93	0.14	0.42
8	116.34	0.23	0.34
9	51.35	0.96	0.08
10	109.58	2.77	4.01
11	236.44	0.95	1.82
12	172.83	1.41	0.21
13	177.31		
14	211.36	0.13	0.23
15	67.22	0.07	0.50
16	112.19	1.79	
17	146.98	0.42	0.45
18	8.56	0.63	0.42
19	193.37	0.33	0.33
20	80.91	3.08	0.72
21	185.18	0.53	0.27
22	74.51	0.10	2.32
23	20.41	3.05	0.22
24	70.36	0.12	0.16
25	85.66	0.14	0.35
26	155.36	0.21	2.26
27	101.06	0.93	1.67
28	70.73		
29	84.90	0.84	2.41
30	139.12		
31	545.66	5.46	3.24
32	248.62	0.20	0.00
33	250.16	0.24	1.58
34	292.43	0.05	0.11

Supplementary figure S1. EVs isolation from plasma - particle/protein ratio

Isolation of EVs from plasma was performed as described in Materials & Methods using 3 homemade columns (prepared 1 day before) and one new commercial Izon qEV/70 nm column. Fractions 1–8 (1 mL each) from the SEC columns were collected and measured for particle content by the NTA method and for protein content by the BCA method (Pierce). Most enriched in particles were fractions 4, 5 and 6 (mean concentration ± SD was: F4: 5.40 x 10^{10} ± 5.13 x 10^{9}; F5: 1.70 x 10^{11} ± 6.17 x 10^{10}; F6: 7.55 x 10^{10} ± 1.79 x 10^{10} particles/mL) (**Fig. S 1a**). The protein concentration was below the detection limit of our BCA method in the first 4 fractions, was low in fractions 5-6, and increased rapidly in fractions 7-8 (**Fig. S 1b**). The particle/protein ratio was most beneficial (highest) in fractions 5 and 6 (ratio ± SD was: F5: 8.40 x 10^{9} ± 1.53 x 10^{9}; F6: 8.42 x 10^{8} ± 1.01 x 10^{8} particles/mL) (**Fig. S 1c**).

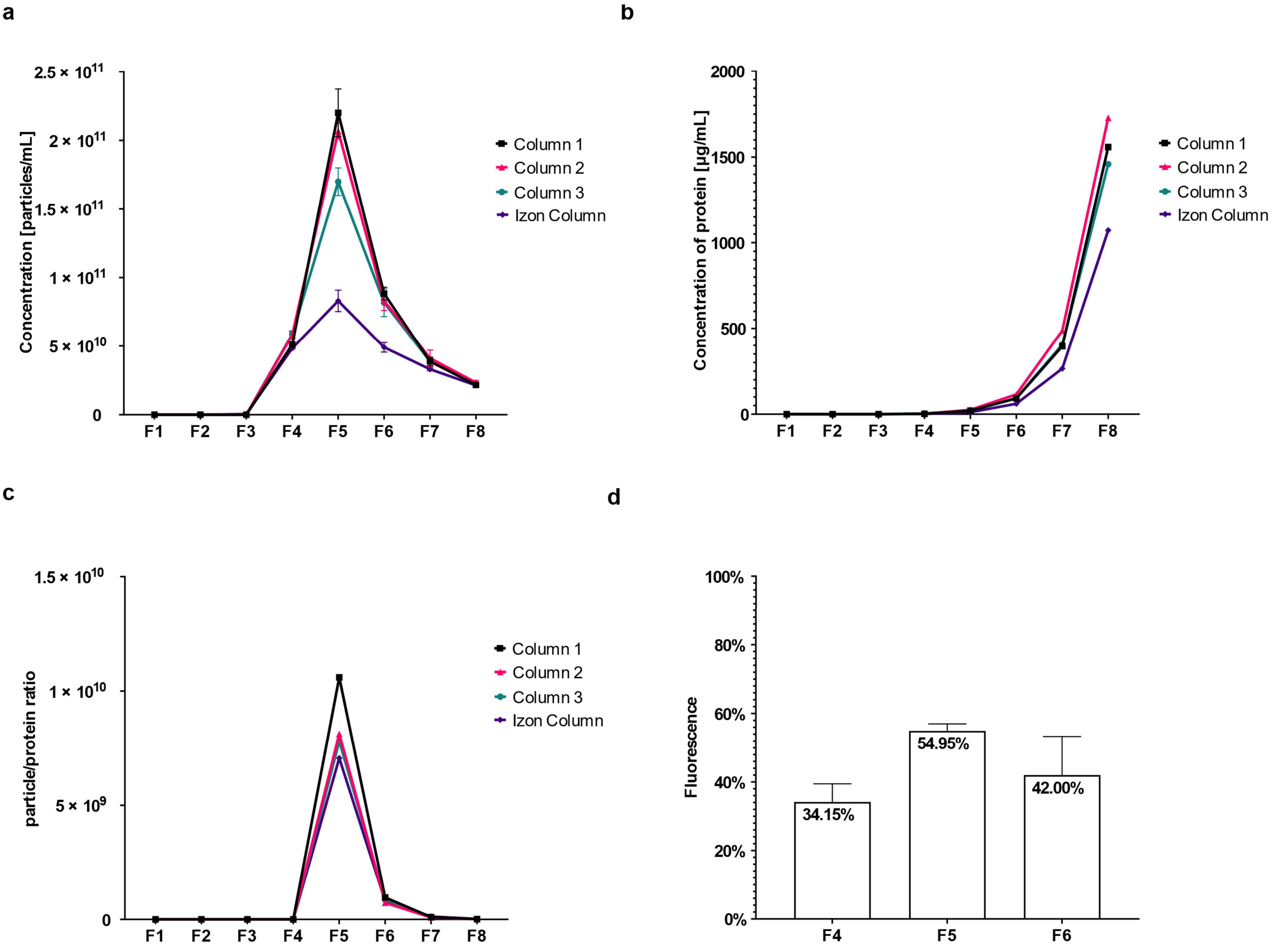

Fig. S1. EVs separation from plasma on SEC - particle/protein ratio. (**a**) The concentration of particles in fractions 1-8 from 3 home-made SEC columns and one commercial Izon column after separation of EVs from plasma. (**b**) The concentration of protein measured in 1-8 fraction for each column. (**c**) Particle/protein ratio in each fraction. (**d**) CMDR positive particles' fluorescence from all visible in the scatter of plasma EVs in fractions 4,5 and 6 from SEC. The experiment from figures (**a-c**) was conducted on two biological samples with three replicate measurements each. Graph d presents Mean and SD from two replicates. The experiment from graph (**d**) was created from measurements taken at Sensitivity 93.

Those results led us to further investigations on fractions 4, 5, and 6. We have performed membrane labeling of fractions 4, 5 and 6. The results showed that in F4 there was 34.15%, in F5 54.95%, and in F6 42.00% CMDR positive particles of all visible particles in scatter (**Fig. S1d**). All three fractions were enriched in EVs, but higher concentration of particles, the measurable concentration of protein, and higher percent of CMDR positive

particles led us to choose fractions 5 and 6, which we pulled for further analysis of the NSCLC patient's plasma-EV samples.

Supplementary figure S2. Flow cytometry of BALF-EVs after coupling to CD63-, CD9- and CD81-specific magnetic beads

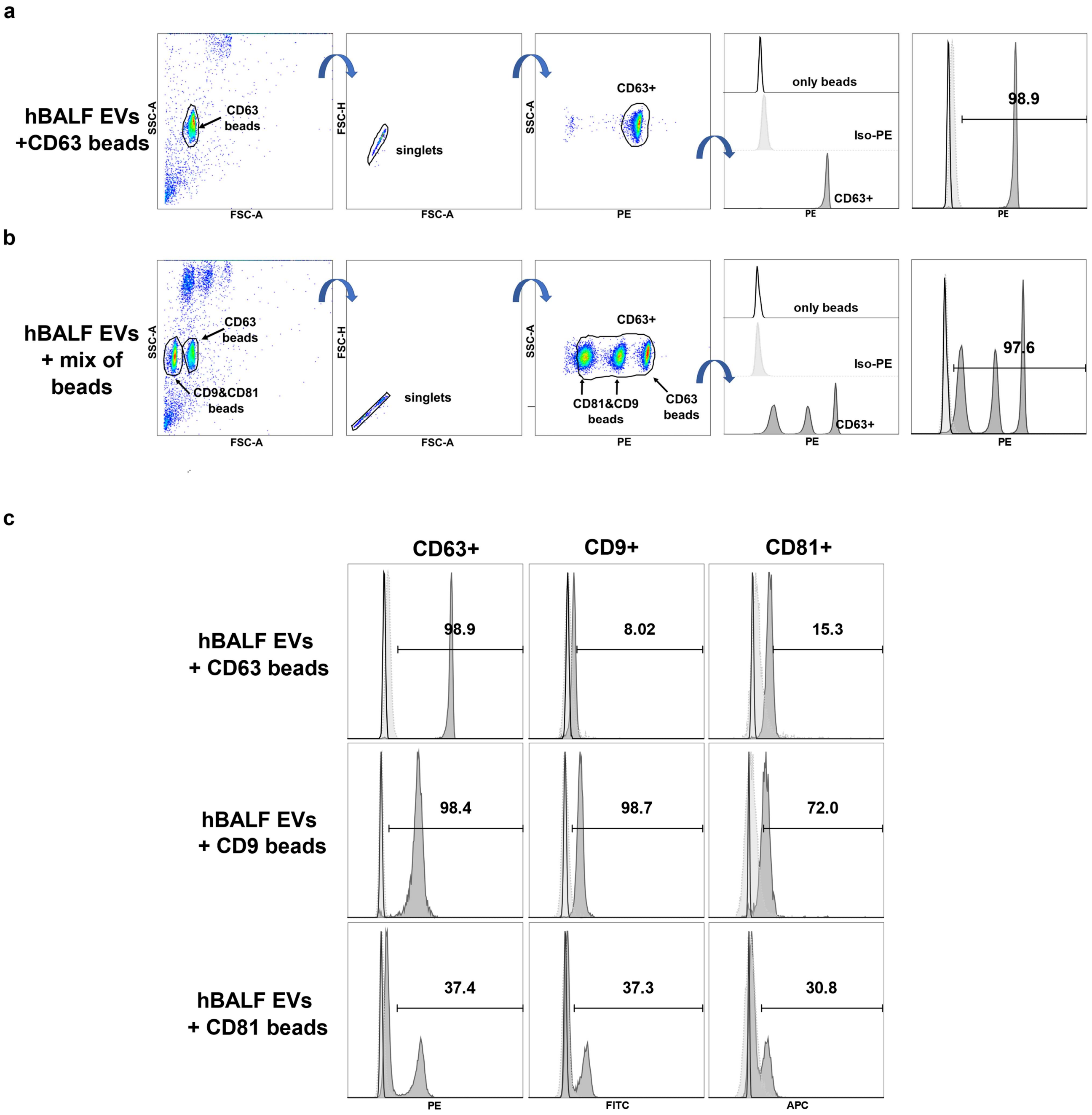

Fig. S2. Flow cytometry analysis of oBALF-EVs bound to magnetic Dynabeads coated with antibodies against tetraspanins. Figures (**a & b**) present examples of gating strategy and overlay creation. Gates were set first on single beads visible in the forward and side scatter, then on singlets. The gate for tetraspanin-positive events was set based on isotype control labeling so that a maximum of 5% of the isotype control was included in the gate. Samples with beads only, isotype labeled sample, and tetraspanin labeled sample were overlayed. (**a**) CD63 beads. (**b**) A mix of CD63, CD9, and CD81 beads. (**c**) One type of beads (CD63, CD9, or CD81) bound to oBALF-EVs and labeled with CD63-PE, CD9-FITC, and CD81-APC. The highest percentage and MFI for a given tetraspanin were usually achieved with beads specific for this given tetraspanin. An exemption were CD81-beads, where only up to 40% of beads captured any vesicles. EVs captured by CD9 beads showed the highest percent for all three markers (98.4% CD63, 98.7% CD9, and 72% CD81 positive particles). In the case of the CD63 beads, the highest percent had CD63 positive particles (98.9%), whereas there were only 8.02% CD9 and 15.3% CD81 positive particles. CD81 beads showed 37.4% of CD63, 37.3% CD9 and 30.8% CD81 positive particles.

Supplementary figure S3. Analytical variability of the NTA measurements

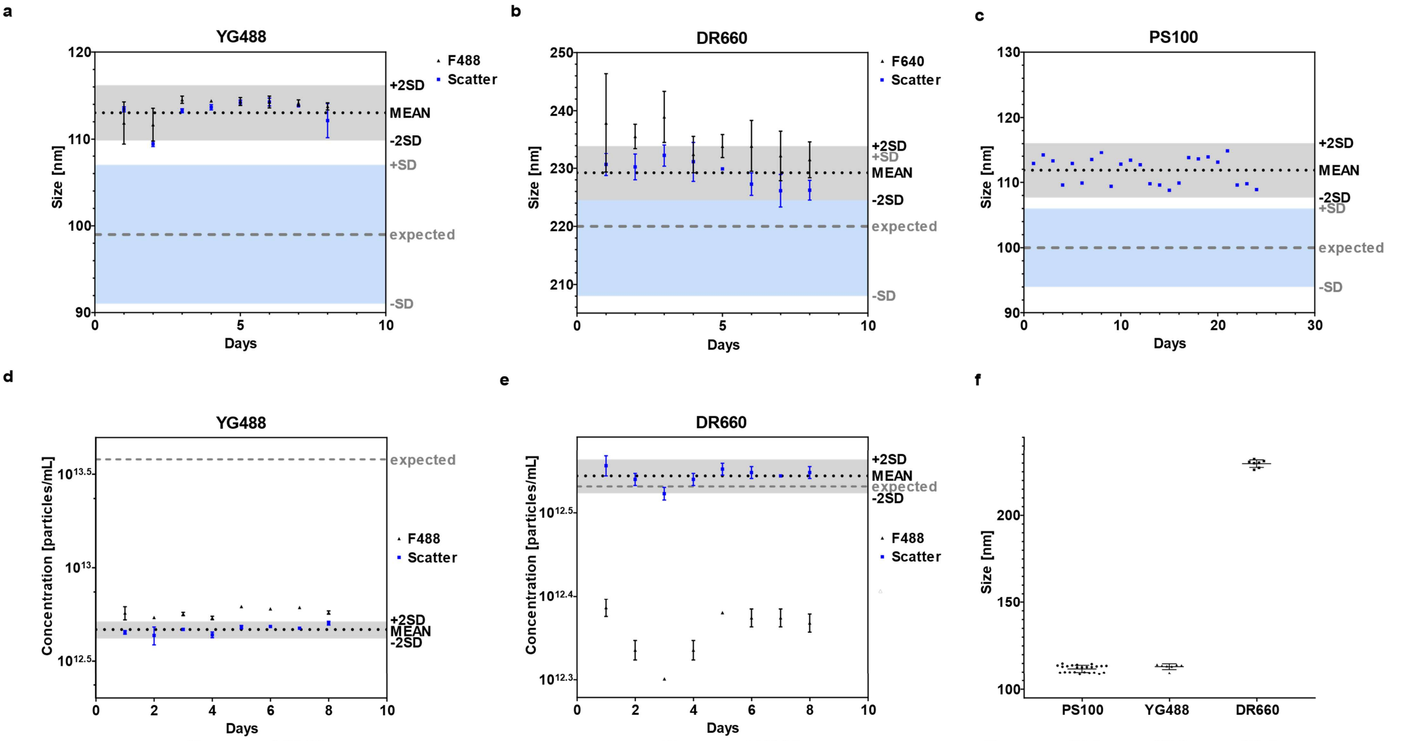

d

Mean	4.66E12
SD	2.43E11
SEM	8.59E10
CV	5.2%

e

Mean	3.50E12
SD	8.16E11
SEM	2.89E10
CV	2.3%

f

	PS100	YG488	DR660
Mean	111.9	113.0	229.0
SD	2.1	1.6	2.3
SEM	0.4	0.6	0.8
CV	1.9%	1.4%	1.0%

Fig. S3. Variability of the measured size and concentration of standard beads on NTA. (**a**) The size of YG488 particles in F488 (black) and scatter (blue) mode. (**b**) The size of DR660 particles in (black) and scatter (blue). (**c**) The size of PS100 particles in scatter mode - single data from calibration beads from 24 days. (**d**) The concentration of YG488 particles in (black) and scatter (blue). (**e**) The concentration of DR660 particles in F660 (black) and scatter (blue). (**f**) The mode size of PS100, YG488, and DR660 in the scatter mode with calculated Mean, SD, SEM, and CV. Graphs (**a, b, d, e**) were prepared from 3 replicate measurements performed for 8 days. Graphs (**a, b, d, e**) present the mean and SD of each replicate. MEAN ± 2 SD area is colored grey. MEAN value is marked with the black dotted line. The expected value from the beads certificate is marked with the grey dotted line (expected) with ± SD area colored blue. All graphs were created from measurements in settings described in Materials & Methods dedicated to each type of beads.

Supplementary figure S4. Optimization of dye concentration for measurements

Staining mixtures were prepared by adding different amounts of CMDR (final concentration for NTA measurement 0.5/1/2/4/6/8/10 [ng/mL]) to a constant appropriate amount of EVs sample (optimal for NTA measurement after final dilution). Mixtures were filled with PBS to a total volume of 10 µl. Samples were incubated for 2 h at RT in the dark. Then, the samples were diluted in PBS and measured on NTA. The optimization experiment was conducted on nonconcentrated (pulled fraction 5 and 6) and concentrated plasma-EVs (pulled fraction 4-6) from Healthy Normal Control and on BALF-EVs from NSCLC patient (**Fig. S4**).

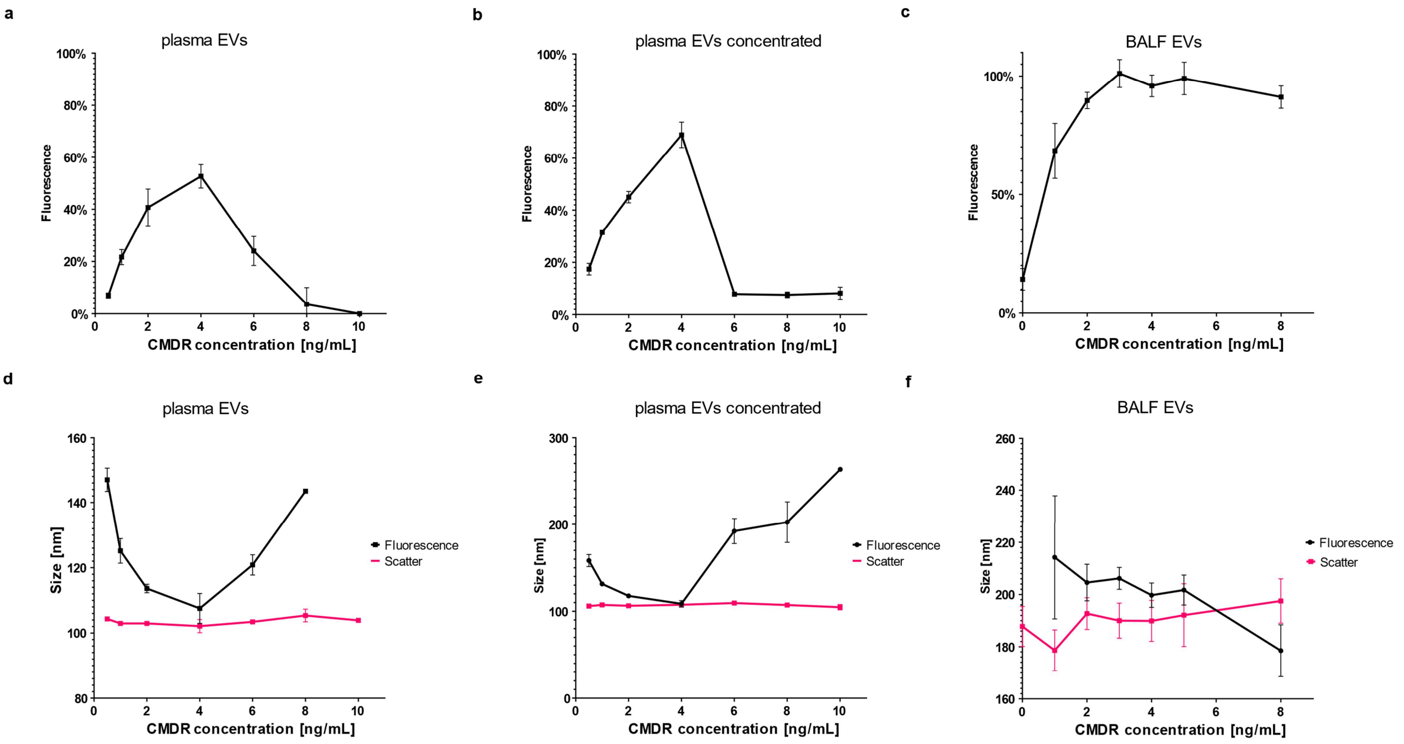

Fig. S4. Optimization of CMDR concentration. (**a-c**) CMDR positive particles' fluorescence from all visible in scatter for fresh, pulled fraction 5 and 6 of plasma EVs (**a**), concentrated, thawed, pulled fractions 4-6 of plasma EVs (**b**), and BALF EVs (**c**). (**d-f**) The mode size of particles in scatter and fluorescence for fresh, pulled fraction 5 and 6 of plasma EVs (**d**), concentrated, thawed, pulled fractions 4-6 of plasma EVs (**e**), and BALF EVs (**f**). Experiments were conducted on 3-6 replicate measurements. Graphs (**a-f**) present Mean and SD. Graphs (**a**) and (**d**) were created from measurements in settings described in Materials & Methods. Graphs (**b, c, e, f**) were created from measurements taken at Sensitivity 93.

The results showed that in plasma-EVs (both concentrated and nonconcentrated), the fluorescence reaches the highest percent for 4 ng/mL of final CMDR concentration on NTA (nonconcentrated: 52.67 ± 4.51%, concentrated:68.9 ± 4.90%), and then drops when more CMDR is present (**Fig. S4a, b**). For plasma-EVs the difference between particles' size in fluorescent and scatter mode was the smallest at 4 ng/mL CMDR (Δ mean size: nonconcentrated:5.42 nm, concentrated:1.1 nm) (**Fig. S4d, e**). In the case of BALF-EVs, the concentration of particles in fluorescent mode reached a plateau at concentration 3 ng/mL CMDR (**Fig. S4c**). The difference between particles' size in fluorescent and scatter mode was the smallest at 2-5 ng/mL CMDR (**Fig.S4f**). The concentration 4 ng/mL CMDR was chosen as optimal for further staining experiments.

Supplementary figure S5. Antibody labeling of cBALF and oBALF EVs

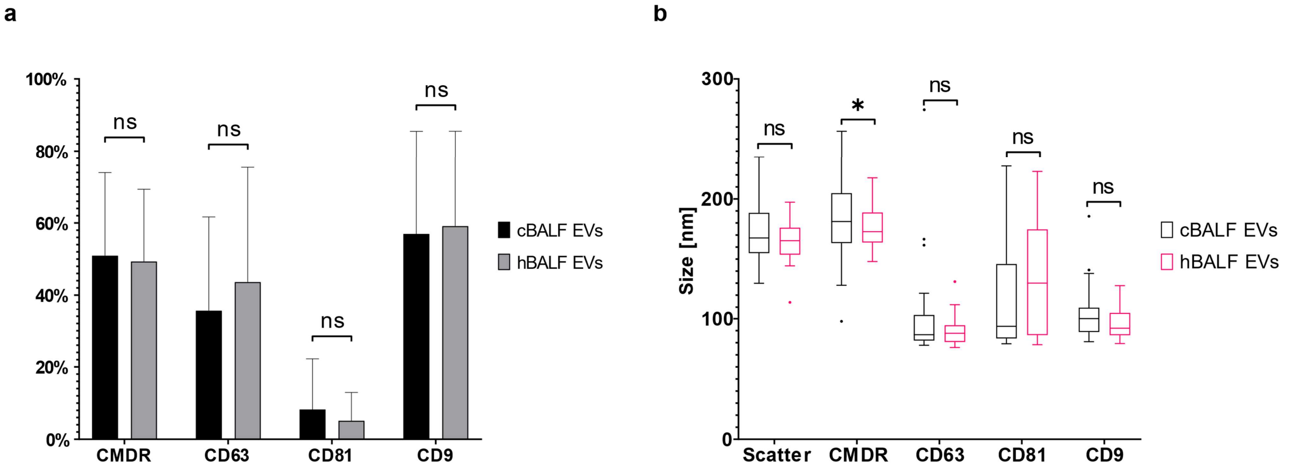

Fig. S5. Antibody labeling of cBALF and oBALF EVs. (**a**) The percent of fluorescent particles in comparison to all particles visible in scatter mode for cBALF and oBALF EVs for all patients. (**b**) Measured mode sizes of particles in scatter and in fluorescent mode (488, 640 nm) for cBALF and oBALF EVs for all patients. Graph (**a**) present Mean and SD for all patients. Graph (**b**) presents the Tukey plot for all patients. * refers to p value ≤ 0.05, ns refers to p value > 0.05 from Wilcoxon test-paired comparison.

Supplementary figure S6. RIPA lysis of NSCLC Cell line EVs - size

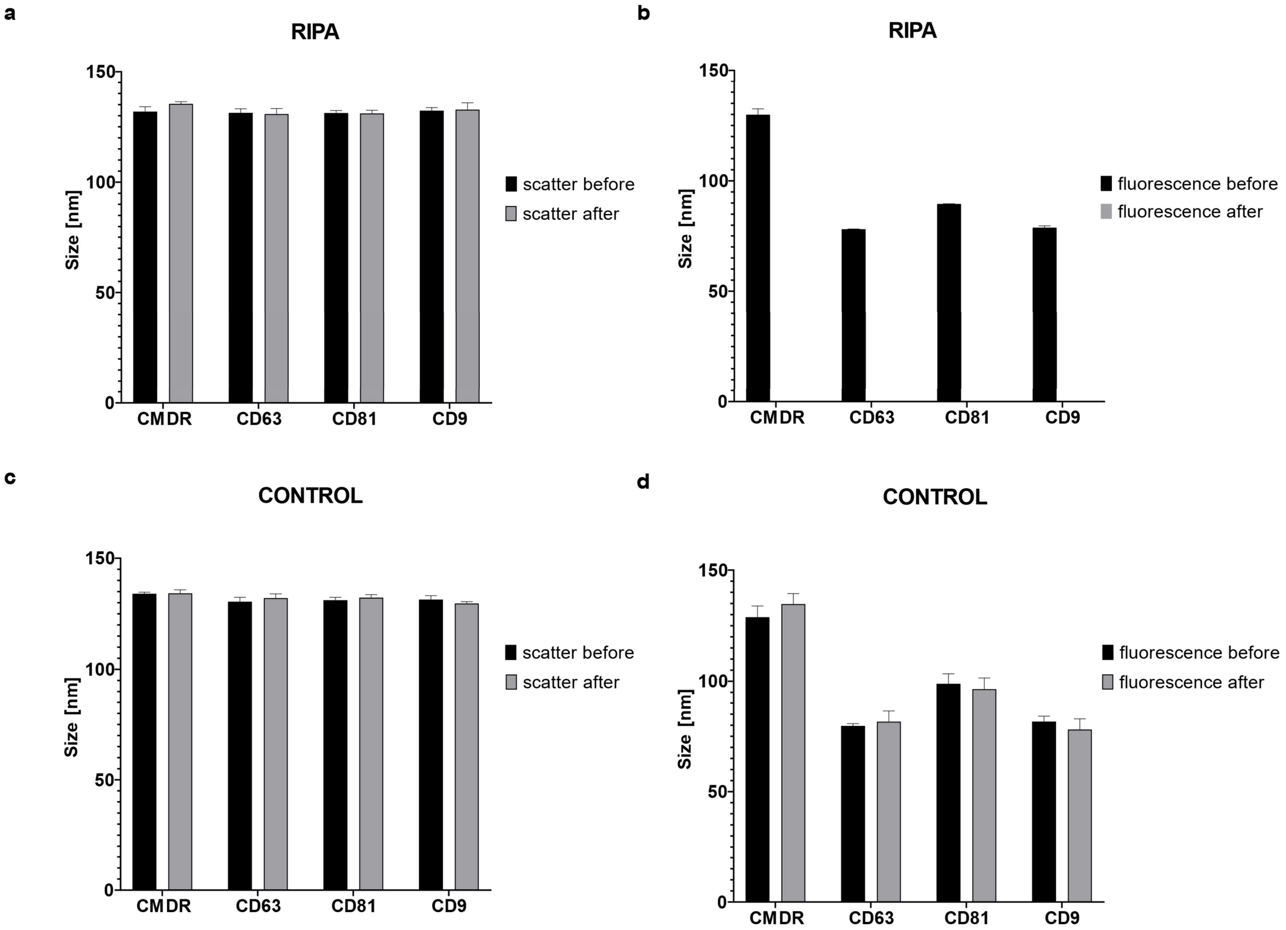

Fig. S6. RIPA lysis of EVs. (**a-b**) Particles' mode size after labeling with CMDR and tetraspanin markers of NSCLC Cell line EVs before and after incubation with RIPA lysis buffer measured in the scatter mode (**a**) and fluorescent mode (**b**). (**c-d**) Particles' mode size after labeling with CMDR and tetraspanin markers of NSCLC Cell line EVs before and after incubation with PBS (Control) measured in the scatter mode (**c**) and fluorescent mode (**d**). Graphs a-d present Mean and SD from three replicates.

Supplementary figure S7. Cryo-TEM images of thawed cBALF EVs

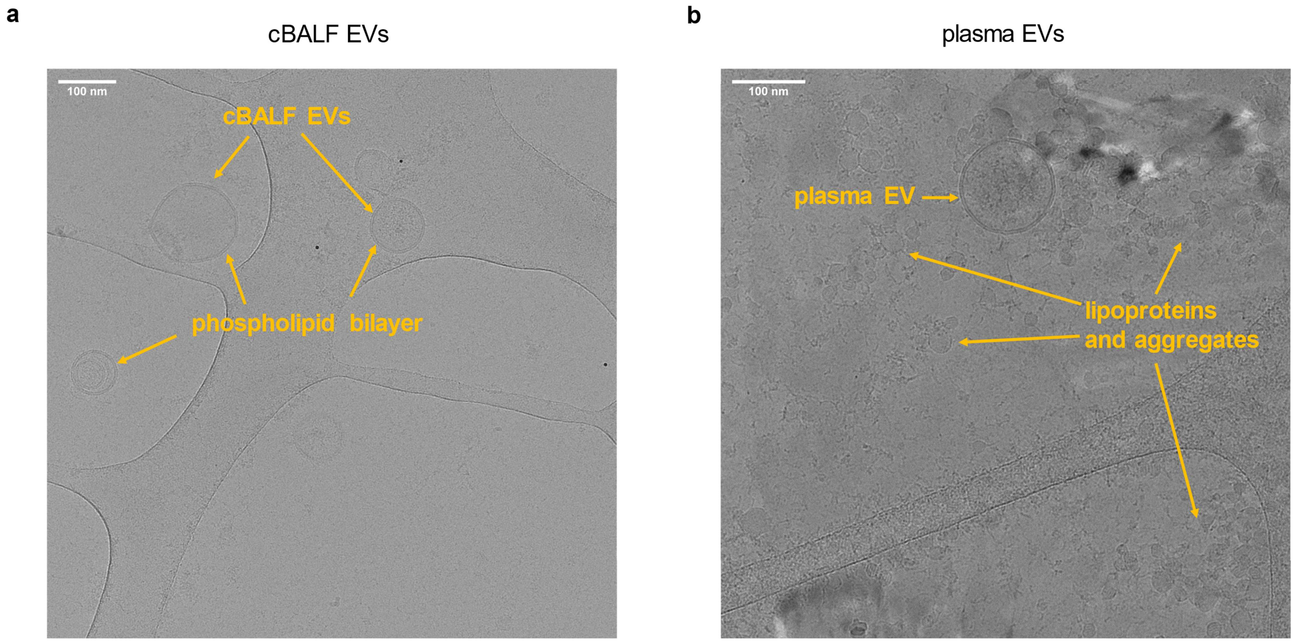

Fig. S7. Cryo-TEM images of thawed cBALF EVs. (**a**) Thawed cBALF EVs. There is a visible double phospholipid bilayer. (**b**) Plasma EVs. Besides EVs, particles with different morphology, like lipoproteins and aggregates, are visible.

Full blots from Fig.2 (a)

Merged (chemiluminescence and colorimetric) blots with described protein ladder: Characterization of plasma/BAL EVs form NSCLC patients. Immunoblot analysis of EVs from Plasma and BALF of two NSCLC patients (Pt.3, Pt.4), a normal donor (NC) and a cell lysate (SEMK2). Each lane with plasma EVs represents the amount of protein present in 100 µl of patients' plasma which was taken for the isolation. Each lane with BAL EVs represents the amount of protein present in 4 ml of Patients' BALF (from healthy or cancer lung) which was taken for the isolation. Cell lysate lane was loaded with 10 µg of protein from cell lysate.

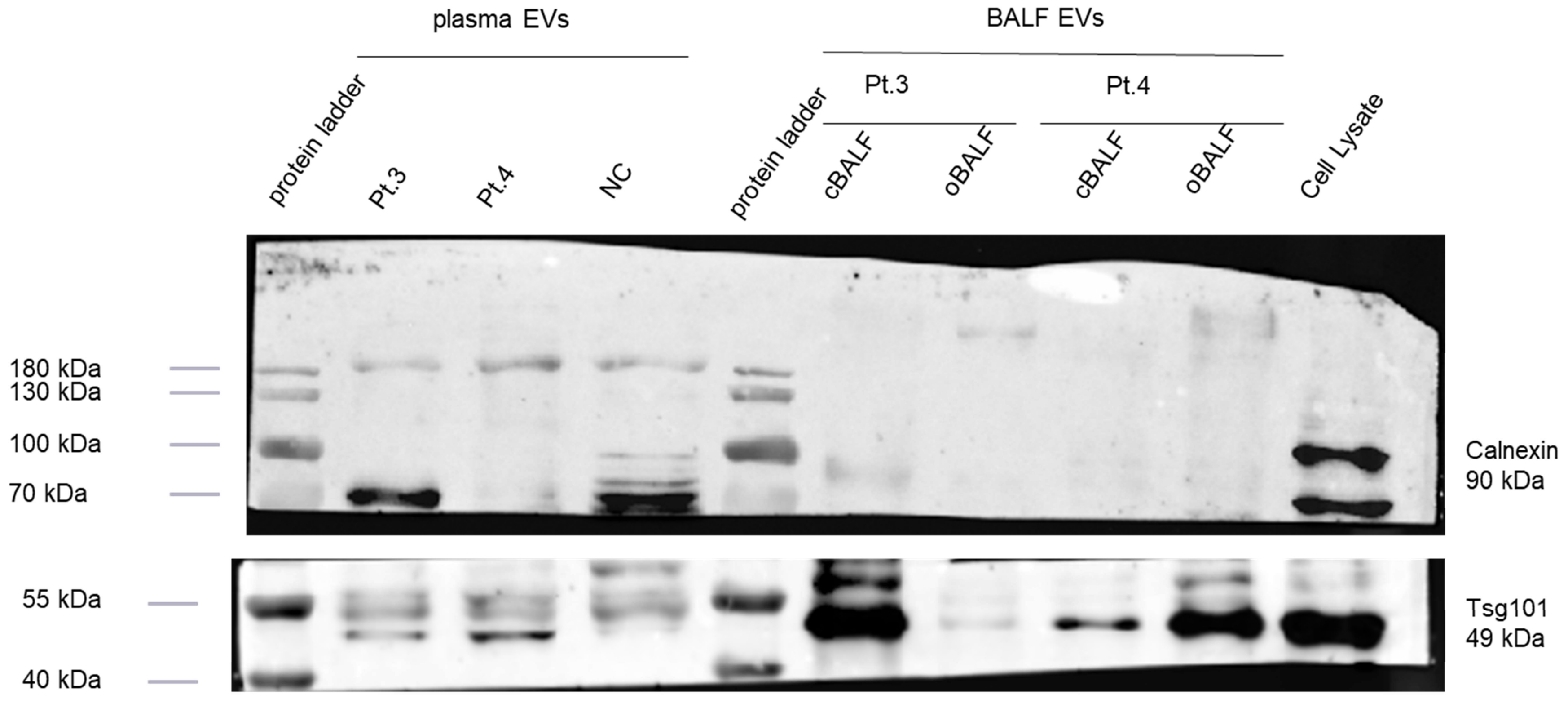

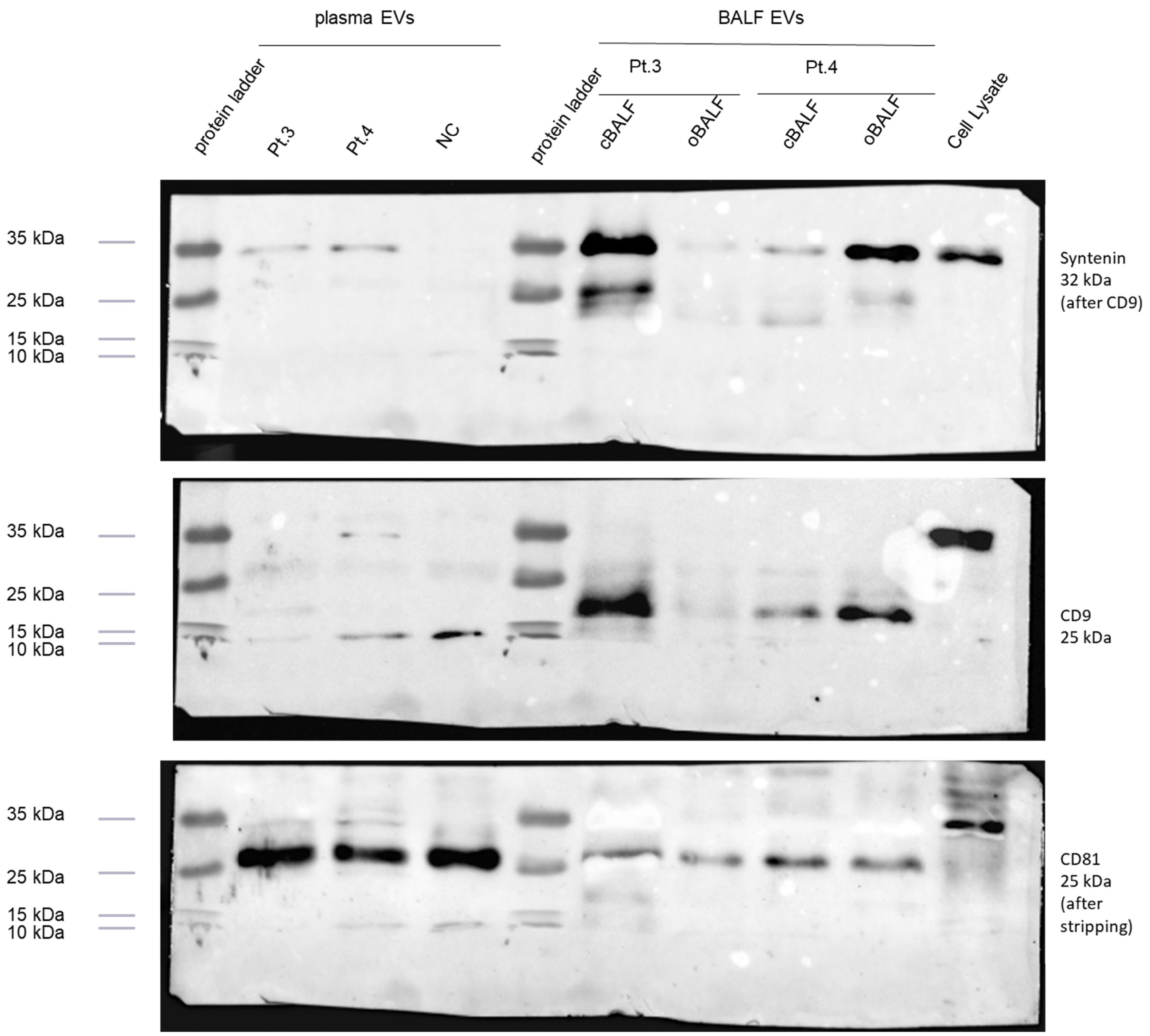
plasma EVs
BALF EVs
Pt.3
Pt.4
protein ladder
Pt.3
Pt.4
NC
protein ladder
cBALF
oBALF
cBALF
oBALF
Cell Lysate
35 kDa
25 kDa
15 kDa
10 kDa
Syntenin
32 kDa
(after CD9)
35 kDa
25 kDa
15 kDa
10 kDa
CD9
25 kDa
35 kDa
25 kDa
15 kDa
10 kDa
CD81
25 kDa
(after
stripping)

Supplementary figure S8. Lipoprotein marker Apo-B

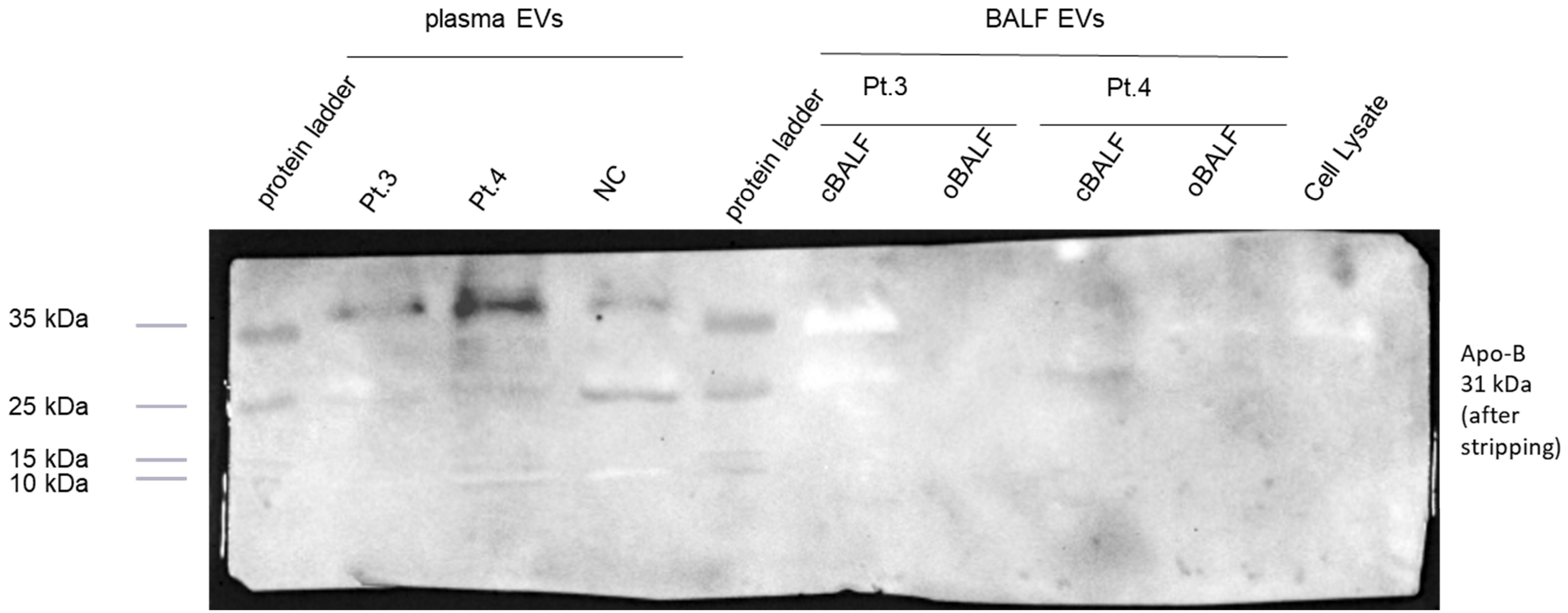

Raw pictures from Cryo-TEM from Fig. 2 (b)

cBALF
EVs

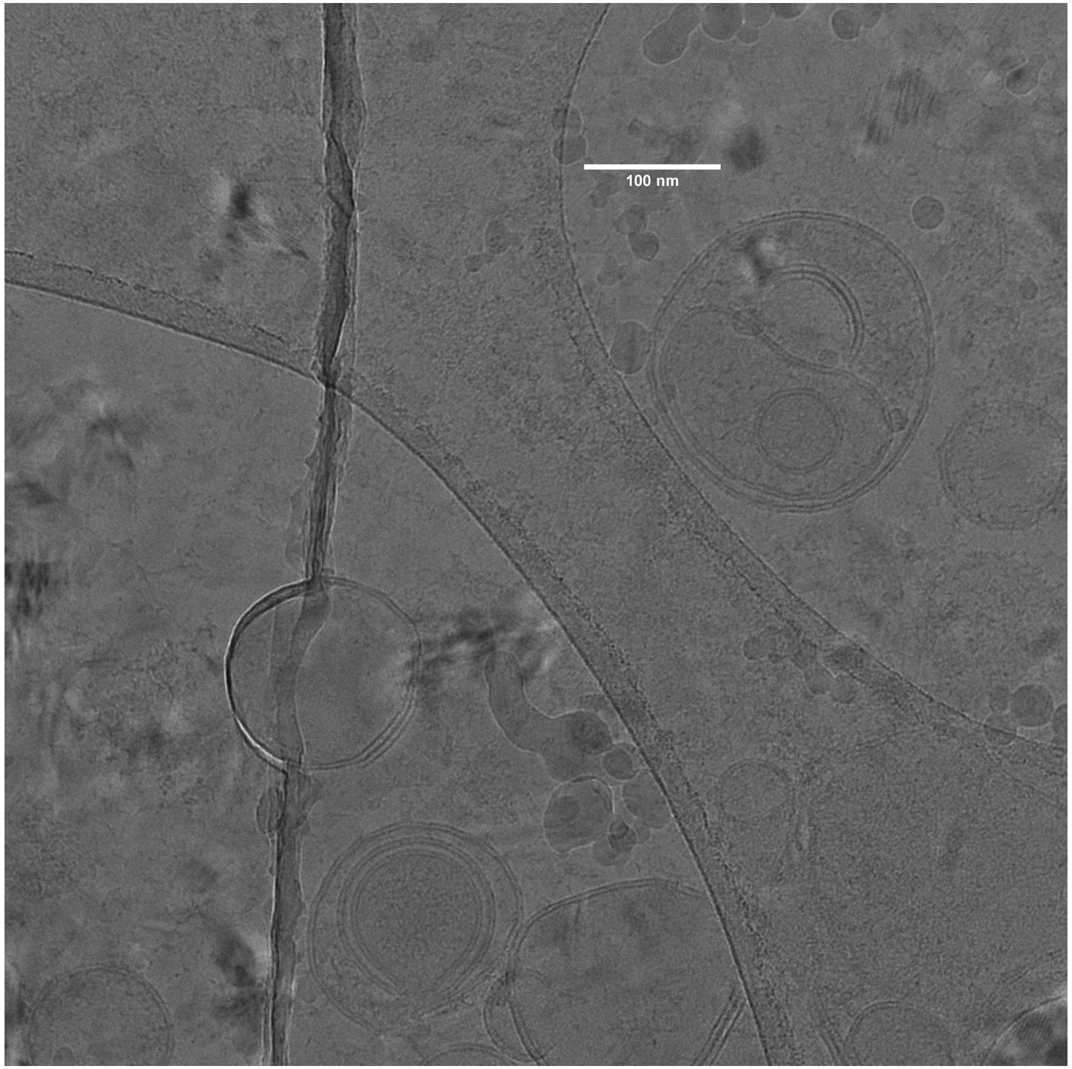

oBALF
EVs

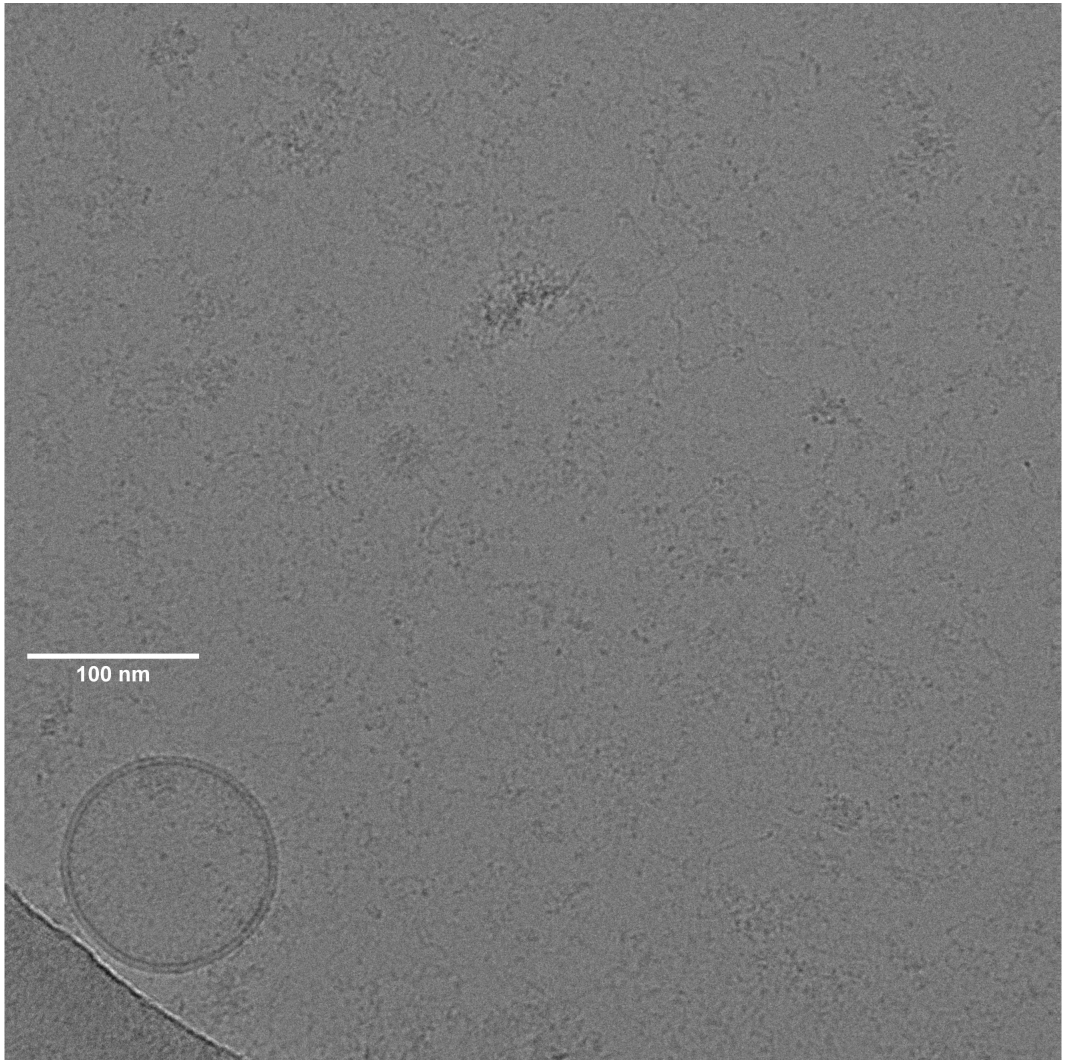

plasma
EVs

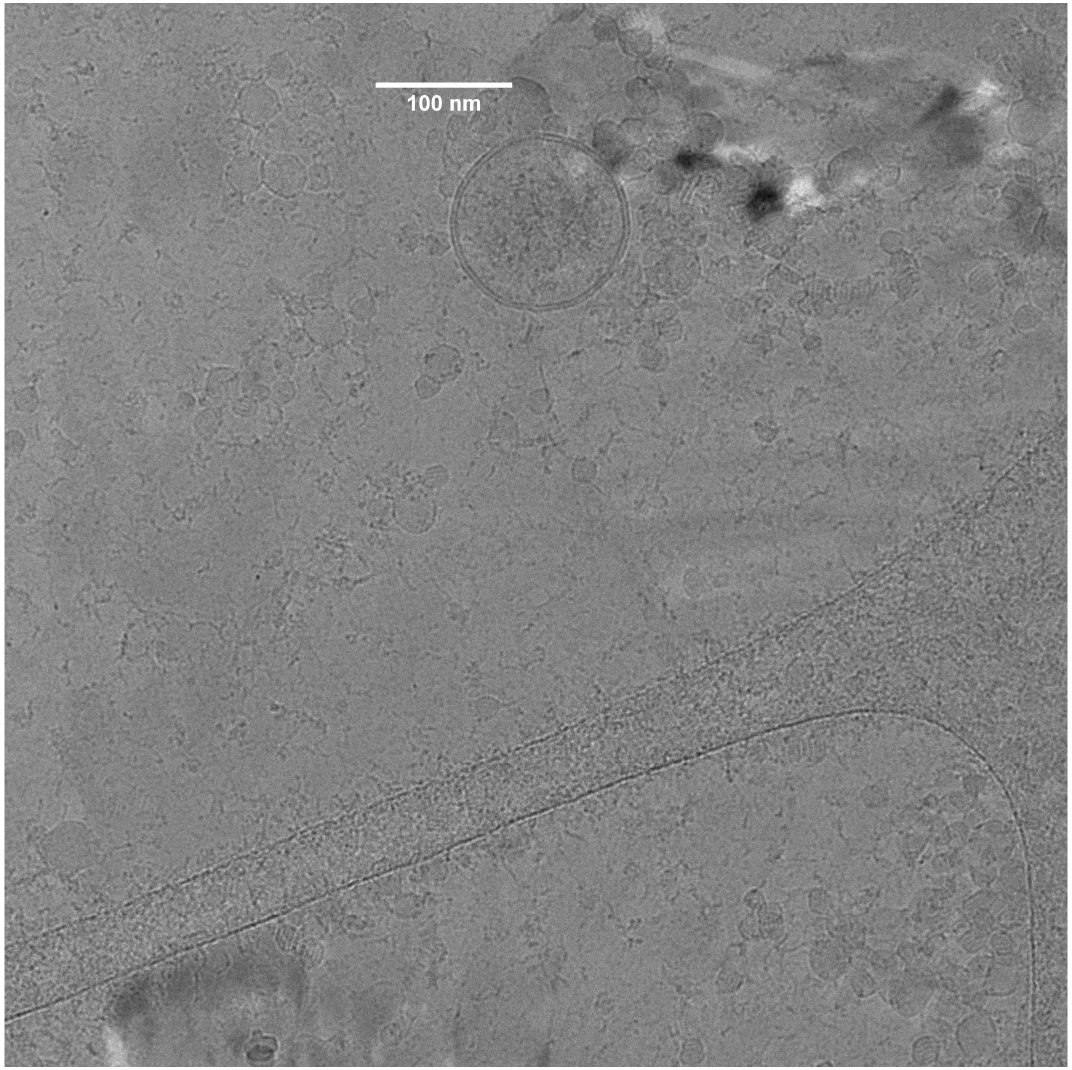

Supplement: Supplementary file 1 [file cells-10-03473-s001.zip › cells-1446798-supplementary.pdf]
